# Episodic disability questionnaire (EDQ) measurement properties among adults living with HIV in Canada, Ireland, United Kingdom, and United States

**DOI:** 10.1186/s12879-023-08958-7

**Published:** 2024-01-10

**Authors:** Kelly K. O’Brien, Kristine M. Erlandson, Darren A. Brown, Soo Chan Carusone, Jaime H. Vera, Colm Bergin, Lisa Avery, Ahmed M. Bayoumi, Steven E. Hanna, Richard Harding, Patricia Solomon, Natalie St. Clair-Sullivan, Noreen O’Shea, Carolann Murray, Marta Boffito, George Da Silva, Brittany Torres, Kiera McDuff, Aileen M. Davis

**Affiliations:** 1 Department of Physical Therapy, Temerty Faculty of Medicine, University of Toronto, 160-500 University Avenue, Toronto, ON M5G 1V7 Canada; 2 Institute of Health Policy, Management and Evaluation (IHPME), Dalla Lana School of Public Health, University of Toronto, Toronto, ON Canada; 3 Rehabilitation Sciences Institute (RSI), University of Toronto, Toronto, ON Canada; 4 University of Colorado Denver-Anschutz Medical Campus, Aurora, CO USA; 5 Chelsea and Westminster Hospital NHS Foundation Trust, London, UK; 6 McMaster Collaborative for Health and Aging, McMaster University, Hamilton, ON Canada; 7 Casey House, Toronto, ON Canada; 8 Department of Global Health and Infection, Brighton and Sussex Medical School, University of Sussex, Brighton, UK; 9 GUIDE Clinic, St. James’s Hospital, Hospital, Dublin, Ireland; 10 School of Medicine, Trinity College Dublin, Dublin, Ireland; 11 Biostatistics Department, Princess Margaret Cancer Centre, University Health Network, Toronto, ON Canada; 12 MAP Centre for Urban Health Solutions, Li Ka Shing Knowledge Institute, St. Michael’s Hospital, Toronto, ON Canada; 13 Department of Medicine, University of Toronto, Toronto, ON Canada; 14 Division of General Internal Medicine, St. Michael’s Hospital, Toronto, ON Canada; 15 Health Research Methods, Evidence and Impact, McMaster University, Hamilton, ON Canada; 16 Florence Nightingale Faculty of Nursing Midwifery and Palliative Care, Cicely Saunders Institute, King’s College London, London, UK; 17 School of Rehabilitation Science, McMaster University, Hamilton, ON Canada

**Keywords:** HIV, Disability evaluation, Questionnaires, Measurement, Reliability, Validity

## Abstract

**Background:**

The Episodic Disability Questionnaire (EDQ) is a generic 35-item patient-reported outcome measure of presence, severity and episodic nature of disability. We assessed the measurement properties of the Episodic Disability Questionnaire (EDQ) with adults living with HIV.

**Methods:**

We conducted a measurement study with adults living with HIV in eight clinical settings in Canada, Ireland, United Kingdom, and United States. We electronically administered the EDQ followed by three reference measures (World Health Organization Disability Assessment Schedule; Patient Health Questionnaire; Social Support Scale) and a demographic questionnaire. We administered the EDQ only 1 week later. We assessed the internal consistency reliability (Cronbach’s alpha; > 0.7 acceptable), and test–retest reliability (Intra Class Correlation Coefficient; > 0.7 acceptable). We estimated required change in EDQ domain scores to be 95% certain that a change was not due to measurement error (Minimum Detectable Change (MDC95%)). We evaluated construct validity by assessing 36 primary hypotheses of relationships between EDQ scores and scores on the reference measures (> 75% hypotheses confirmed indicated validity).

**Results:**

Three hundred fifty nine participants completed the questionnaires at time point 1, of which 321 (89%) completed the EDQ approximately 1 week later. Cronbach’s alpha for internal consistency ranged from 0.84 (social domain) to 0.91 (day domain) for the EDQ severity scale, and 0.72 (uncertainty domain) to 0.88 (day domain) for the EDQ presence scale, and 0.87 (physical, cognitive, mental-emotional domains) to 0.89 (uncertainty domain) for the EDQ episodic scale. ICCs for test–retest reliability ranged from 0.79 (physical domain) to 0.88 (day domain) for the EDQ severity scale and from 0.71 (uncertainty domain) to 0.85 (day domain) for the EDQ presence scale. Highest precision was demonstrated in the severity scale for each domain (MDC95% range: 19–25 out of 100), followed by the presence (MDC95% range: 37–54) and episodic scales (MDC95% range:44–76). Twenty-nine of 36 (81%) construct validity hypotheses were confirmed.

**Conclusions:**

The EDQ possesses internal consistency reliability, construct validity, and test–retest reliability, with limited precision when administered electronically with adults living with HIV across in clinical settings in four countries. Given the measurement properties, the EDQ can be used for group level comparisons for research and program evaluation in adults living with HIV.

**Supplementary Information:**

The online version contains supplementary material available at 10.1186/s12879-023-08958-7.

## Background

People with Human Immunodeficiency Virus (HIV) are living longer and may be aging with health-related challenges related to other health conditions in addition to HIV, often referred to as disability [1–3]. Disability is defined as any health related challenge, where health challenges may persist, or fluctuate on a daily basis or over the longer course of living with HIV [4, 5].

Measuring disability is important for identifying and determining the impact of health challenges, improving communication between providers and patients, and evaluating the effect of interventions [6–8]. We developed the Episodic Disability Questionnaire (EDQ) a patient-reported outcome measure of disability, derived from an HIV-specific measure of disability, the HIV Disability Questionnaire (HDQ) developed to describe disability experienced by adults living with HIV [9]. The EDQ is based on a conceptual framework of disability [4, 10], and the foundational HDQ, which possessed validity, reliability, and sensibility for use among adults living with HIV in Canada, Ireland, the United States (US), and United Kingdom (UK) [11–15]. However, at 69 items, the HDQ was lengthy posing challenges for clinical use [16].

We shortened the HDQ to a 35-item version Short-Form HIV Disability Questionnaire (SF-HDQ), spanning six domains: i) physical; ii) cognitive; iii) mental-emotional health challenges, iv) uncertainty or worrying about the future, v) difficulties carrying out day-to-day activities, and vi) challenges to social inclusion [17]. The SF-HDQ possessed sensibility and utility for use with adults living with HIV in clinical and community-based settings in Canada, Ireland, US, and UK [18]. However, adults living with HIV and HIV health care practitioners questioned the need to specify HIV as the source of health challenges for some SF-HDQ items related to uncertainty in finance and housing, and social inclusion [18]. Attributing specific health challenges to HIV may be difficult for individuals, as challenges may not be directly due to HIV, but rather attributed to side effects from treatments, aging or concurrent health conditions [18]. Hence, we removed the HIV specificity of items and instructions in the SF-HDQ to establish a new generic measure of episodic disability, renamed the EDQ. The EDQ has the potential to describe the multi-dimensional and episodic nature of disability, regardless of its source, broadening the potential applicability for use with other health conditions. Despite a similar domain structure, the psychometric properties of the EDQ, when administered electronically across different clinical contexts, with adults living with HIV were unknown.

Our aim was to assess the measurement properties of the newly established EDQ for its ability to measure the presence, severity and episodic nature of disability among adults living with HIV in Canada, Ireland, United Kingdom, and United States.

## Methods

We conducted a cross-sectional measurement study involving administration of the EDQ and criterion measures with adults living with HIV in Canada, United Kingdom, Ireland and United States. We followed the (COnsensus‐based Standards for the selection of health Measurement Instruments (COSMIN) guidelines for assessment and reporting of psychometric properties of the EDQ [19–23].

### Study setting

This study was conducted at eight clinical settings, in five cities, in four countries: Canada (Casey House, Toronto), Ireland (Department of Genitourinary Medicine and Infectious Diseases (GUIDE), St. James’s Hospital, Dublin), the United States (The University of Colorado Infectious Diseases Group Practice Clinic, University of Colorado, Denver), and United Kingdom (Royal Sussex County Hospital, Brighton, and Chelsea and Westminster NHS Foundation Trust, London involving four sites: Kobler Rehabilitation Clinic, 10 Hammersmith Broadway, 56 Dean Street, and West Middlesex University Hospital). The Dublin, Denver, and UK sites are HIV outpatient clinics, and the Toronto site is a specialty HIV hospital including an inpatient and day health program for people living with and at risk of HIV.

We received ethics approval from the University of Toronto (Protocol # 38152), University of Colorado (Protocol # 19–1895), St. James’s Hospital (Protocol # 2019–12), London Fulham Research Ethics Committee (REC reference: 20/LO/0909) and NHS Health Research Authority (IRAS project ID: 284075).

### Participants

We recruited adults (18 years of age or older) living with HIV from each site using a recruitment poster asking interested individuals to contact the local study investigator. Informed consent was obtained from the participants in the study by checking ‘yes I consent to participate in the study’ at the initial information and consent page of the questionnaire administration.

### Data collection

We electronically administered the EDQ followed by three criterion measures (World Health Organization Disability Assessment Schedule (WHO-DAS 2.0) [24, 25], Patient Health Questionnaire (8-item) (PHQ-8) [26] and MOS- Social Support Scale) [27] and a demographic questionnaire using the web-based software Qualtrics [28]. Participants completed the questionnaires in-person via a tablet at the clinical site or remotely via a link in an email or Short Message Service (SMS) text. We administered the EDQ only, again 1 week later. At this time, we asked whether participants had a major change in their health status since their last EDQ completion and if yes, to describe the change in their health.

#### Questionnaires

*Episodic Disability Questionnaire*: The EDQ is a newly developed patient-reported outcome measure (PROM) refined from the SF-HDQ [17], comprised of 35-items spanning the six domains: i) physical (10 items); ii) cognitive (3 items); iii) mental-emotional health challenges (5 items), iv) uncertainty or worry about the future (5 items), v) difficulties carrying out day-to-day activities (5 items), and vi) challenges to social inclusion (7 items). For each item, individuals are asked to indicate to what extent they are living with a given health-related challenge on that day (*severity* scale of 0 to 4), and whether that challenge fluctuated in the past week (*episodic score* yes or no). The *presence* score is derived by dichotomizing severity as present (severity 1–4) or absent (severity of 0). See Additional file 1 for the Episodic Disability Questionnaire (EDQ) items.

*World Health Organization Disability Assessment Schedule*: The WHODAS 2.0 is a 36-item self-administered generic questionnaire of functioning and disability applicable across cultures in adult populations, and directly linked to the International Classification of Functioning, Disability, and Health (ICF) [24, 25]. The WHODAS 2.0 assesses difficulty in performing specific functions over the previous 30 days across six disability domains: i) cognition, ii) mobility, iii) self-care, iv) getting along, v) life activities and vi) participation). Individuals provide an answer for each question on a 5-point Likert scale (range 0–4) with higher scores indicating increasing difficulty completing the task [29]. The WHODAS possesses internal consistency and test–retest reliability and validity and cross-cultural applicability spanning 19 countries [29, 30]. The WHODAS is validated in patients with chronic diseases [31] and people living with HIV [32].

*Patient Health Questionnaire*: The PHQ-8 is an 8-item measure of depression severity. Items are rated using a Likert-type scale from 0–3, with a total score range of 0–24. A score of 10 or greater is considered major depression, 20 or more is severe major depression [26]. The PHQ-8 is reliable and valid for use with people living with HIV [33].

*Social Support Survey Questionnaire*: The MOS-SSS is a self-administered 20-item questionnaire designed to measure five different dimensions of social support among patients with chronic illness (emotional/informational support, tangible support, positive social interaction and affectionate support) using 5 response options ranging “none of the time” to “all of the time.” [27] Higher scores indicate higher levels of social support. The MOS-SSS possesses construct validity and reliability with people living with HIV [34].

*Demographic Questionnaire*: The demographic questionnaire included 26 items comprised of demographic (e.g. age, sex, gender, race), HIV (e.g. date of HIV diagnosis, viral load), and health characteristics (e.g. concurrent health conditions, general health status).

### Analysis

We calculated median (interquartile ranges (IQR)) EDQ scores. Severity and presence domain scores were calculated using the algorithm developed through Rasch analysis (score range: 0–100) [17]. Episodic scores included a simple sum transformed on a scale of 0–100. Higher scores indicated greater presence, severity and episodic nature of disability. We calculated median WHODAS 2.0 domain scores, PHQ-8 scores and MOS-SSS domain scores as per guidelines. For the demographic questionnaire we calculated descriptive statistics including frequencies (%) for categorical variables and median and interquartile ranges (IQR) for continuous variables.

*Internal Consistency Reliability:* We calculated Cronbach’s alpha for time 1 (T1) EDQ domain scores of the severity scale and presence scales with 95% confidence intervals (CIs) (> 0.7 acceptable) [35].

*Test–Retest Reliability*: We calculated Intra Class Correlations (ICCs) with 95% CIs using T1 and time 2 (T2) EDQ scores estimated from Shrout and Fleiss’ ICC (2,1) (lower bound CI of > 0.7 acceptable) [35]. We calculated ICCs with the entire sample of participants who indicated that they did not have a change in their health status. We then estimated ICCs based on mode of administration either remote (independently via SMS or email link) or in-person (tablet). Our test–retest assessment focused on presence and severity scales of the EDQ as the episodic scale refers to fluctuations in disability in the past week, hence we did not expect consistency in this scale.

*Minimum Detectable Change (MDC)*: We estimated MDC for EDQ domain scores with 90% and 95% confidence as follows [36]: $MDC={z}_{1-\alpha /2}{\widehat{\sigma }}_{baseline}\sqrt{2(1-\rho )}$ where: $\rho$ is the test–retest reliability; $1-\alpha /2$ is the level of confidence; and ${\widehat{\sigma }}_{baseline}$ is the standard deviation of the measure at baseline.

*Construct Validity*: We examined correlations for 36 primary a priori hypotheses theorizing relationships between EDQ and the WHODAS 2.0 criterion measure subscales, and EDQ scores and known groups of participants completing the EDQ on a good versus bad day. We examined an additional 44 exploratory a priori hypotheses theorizing relationships between EDQ and the PHQ-8 and MOS-SSS criterion measure subscales, self-rated general health status, and known groups of participants living with ≥ 2 versus ≤ 1 concurrent health conditions in addition to HIV (80 hypotheses total). Our construct validity assessment focused on presence and severity scales of the EDQ as the criterion measures do not capture the episodic nature of disability. We derived the a priori hypotheses from earlier construct validity assessments of the HDQ and SF-HDQ [12–14]. Spearman correlation coefficients of |≥ 0.30|, |≥ 0.50| and |≥ 0.70|, were defined as ‘weak’, ‘moderate’ and ‘strong,’ respectively [37]. We interpreted the lower and upper bound of confidence intervals when assessing the hypotheses. Construct validity was defined as > 75% confirmed hypotheses [35, 38].

Analysis was conducted using R [@R-psych] [39] and SPSS Software [40].

Sample Size: To detect a weak correlation |*r* = 0.20|, between EDQ and criterion scores, with a power of 0.90 and alpha of 0.05 required 259 participants [41]. To account for questionnaires with missing responses and loss to follow-up at T2, our targeted sample size was 75 adults living with HIV in each of the five cities for a total of 375 participants [42].

## Results

Three hundred fifty-nine participants completed the questionnaires at T1, of which 321 (89%) completed the EDQ at T2. Most participants (80%) completed the T2 EDQ within two weeks of T1 completion.

Of the 321 participants who completed T2, 46 (15%) 274 participants (85%) reported no change in health status and were included in test–retest reliability assessment. The characteristics of those who did and did not complete the EDQ at T2 were similar (see Additional file 2).

### Characteristics of participants

See Table 1 of characteristics of participants by site. Most participants identified as men (83%), median age 51 years, living with a median of 4 concurrent health conditions in addition to HIV. There were differences in participant characteristics (Table 1) and EDQ scores across cities, reflecting the types of populations and services provided across the sites. Participants at Casey House, which is a day health program in Toronto tended to be living with more concurrent health conditions, were less likely to be employed, and more likely be on income support compared to participants from the other sites (Table 1). See Additional file 3 for a complete overview of participant characteristic differences across cities. See Additional file 4 for differences in EDQ scores and criterion measure scores across cities.

**Table 1:** Characteristics of participants by city (*n* = 359)

Characteristic	Entire Sample (*n* = 359)	Brighton, UK (*n* = 75)	London, UK (*n* = 75)	Denver, United States (*n* = 78)	Dublin, Ireland (*n* = 51)	Toronto, Canada (*n* = 80)	*p*-value (Statistical Test)
**Age (years) (*n* = 353)**							
Median (25, 75th percentile)	51 (42, 59)	54 (47, 60)	51 (44, 60)	51 (39, 57)	43 (36, 54)	55 (49, 61)	< 0.001 (KW)
**Gender (*n* = 354)**							
Cis-Woman	40 (11%)	1 (1%)	4 (6%)	19 (25%)	9 (18%)	7 (9%)	< 0.001 (MC)
Cis-Man	293 (83%)	74 (99%)	65 (90%)	52 (68%)	41 (82%)	61 (76%)	
Trans Woman: Man to woman	3 (1%)	0 (0%)	0 (0%)	1 (1%)	0 (0%)	2 (3%)	
Two-spirited	11 (3%)	0 (0%)	2 (3%)	3 (4%)	0 (0%)	6 (8%)	
Non-binary	5 (1%)	0 (0%)	1 (1%)	2 (3%)	0 (0%)	2 (3%)	
Other^a^	2 (< 1%)	0 (0%)	0 (0%)	0 (0%)	0 (0%)	2 (3%)	
**Marital or Partnership Status (*n* = 339)**							
Single	196 (55%)	39 (53%)	45 (61%)	42 (54%)	17 (34%)	53 (66%)	0.001 (MC)
Married, common-law, partner or relationship	88 (25%)	15 (20%)	17 (23%)	24 (31%)	24 (48%)	8 (10%)	
Separated or Divorced or Widowed	55 (15%)	15 (20%)	10 (14%)	11 (14%)	5 (10%)	14 (18%)	
**Have Children (*n* = 355)**	86 (24%)	13 (17%)	10 (14%)	28 (36%)	12 (24%)	23 (29%)	0.008 (FE)
**Live alone (*n* = 357)**	186 (51%)	34 (45%)	43 (58%)	32 (41%)	13 (26%)	64 (80%)	< 0.001 (MC)
**Main Source of Income (*n* = 357)**							
Employment (full, part-time, or self) Income Support (e.g. Disability, Welfare,	177 (50%)	50 (67%)	39 (53%)	43 (55%)	41 (82%)	4 (5%)	< 0.001 (MC)
Worker’s Compensation) Employment Insurance or Long Term Disability)	121 (34%)	10 (13%)	24 (32%)	25 (32%)	7 (14%)	55 (69%)	
Pension, Student Loans, or Savings	41 (12%)	13 (17%)	6 (8%)	3 (4%)	1 (2%)	18 (23%)	
Under the table work or Street-related work (e.g. pan-handling)	3 (1%)	0 (0%)	0 (0%)	1 (1%)	0 (0%)	2 (3%)	
**Current employment status (*n* = 353)**							
Employed (full time or part time)	167 (47%)	48 (65%)	35 (47%)	44 (56%)	37 (76%)	3 (4%)	< 0.001 (MC)
Student, Retired, or Volunteering	59 (17%)	15 (20%)	11 (15%)	6 (8%)	5 (10%)	22 (28%)	
Unemployed or on disability	124 (35%)	11 (15%)	26 (35%)	26 (33%)	6 (12%)	55 (69%)	
Other (baby sitter, carer, stay at home parent)	5 (1%)	0 (0%)	2 (3%)	2 (3%)	1 (2%)	0 (0%)	
**Highest level of education (*n* = 357)**							
No formal education; secondary school completed	88 (25%)	15 (20%)	18 (24%)	19 (24%)	11 (22%)	25 (31%)	0.29 (MC)
Completed trade or technical training, or completed college	132 (37%)	27 (36%)	24 (32%)	30 (39%)	16 (32%)	35 (44%)	
Completed university or postgraduate education	137 (38%)	33 (44%)	32 (43%)	29 (37%)	23 (46%)	20 (25%)	
**How do you describe your race?**							
White (*n* = 314)	255 (81%)	64 (87%)	53 (90%)	52 (72%)	36 (80%)	50 (78%)	0.075 (FE)
Black or African American (*n* = 234)	27 (12%)	2 (3%)	12 (27%)	1 (2%)	5 (13%)	7 (14%)	< 0.001 (FE)
Asian (*n* = 227) (origins in far east, south east Asia, or Indian subcontinent including e.g. Cambodia, China, India, Japan, Korea, Malaysia, Pakistan, Philippine Islands, Thailand, Vietnam)	26 (10%)	5 (8%)	7 (18%)	6 (10%)	2 (5%)	6 (13%)	0.391 (FE)
First Nations (Indigenous), Inuit, Métis (*n* = 255)	31 (12%)	0 (0%)	1 (3%)	19 (29%)	0 (0%)	11 (22%)	< 0.001 (FE)
**Year of HIV diagnosis (*n* = 244)**							
Median	2005	2007	2001	2002	2014	2000	< 0.001 (KW)
25, 75th percentile	1997, 2013	1998, 2013	1997, 2001	1992, 2013	2010, 2018	1992, 2007	
**Most recent CD4 count (*n* = 353)**							
< 200 cells/mm^3^	44 (13%)	10 (14%)	6 (8%)	14 (18%)	4 (8%)	10 (13%)	0.013 (MC)
201–499 cells/mm^3^	61 (17%)	17 (24%)	15 (20%)	11 (14%)	2 (4%)	16 (20%)	
> 500 cells/mm^3^	134 (38%)	22 (31%)	23 (31%)	32 (42%)	31 (62%)	26 (33%)	
Don’t know	114 (32%)	23 (32%)	30 (41%)	20 (26%)	13 (26%)	28 (25%)	
**Currently Taking Antiretroviral Medication (*n* = 357)**	351 (98%)	75 (100%)	73 (99%)	77 (99%)	50 (100%)	76 (95%)	0.16 (FE)
**Missed at least one ARV dose in the last 7 days (*n* = 350)**	281 (80%)	66 (88%)	57 (78%)	60 (80%)	45 (90%)	53 (70%)	0.02 (FE)
**Undetectable viral load (< 50 copies/mL) (*n* = 354)**	328 (93%)	73 (99%)	69 (93%)	66 (85%)	72 (91%)	72 (91%)	0.008 (FE)
**Median # of co-morbidities (25, 75th percentile) (*n*** **= 357)**	4 (2, 9)	3 (1, 5)	4 (1, 9)	4 (2, 9)	3 (1, 4)	8 (4, 12)	< 0.001 (KW)
**Common Concurrent health condition (≥ 30% of sample)**							
Mental health condition (*n* = 351)	162 (46%)	22 (30%)	34 (47%)	36 (47%)	16 (33%)	54 (69%)	< 0.001 (MC)
Chronic pain (joint) (*n* = 346)	113 (33%)	13 (18%)	23 (32%)	29 (38%)	4 (8%)	44 (58%)	< 0.001 (MC)
Gastrointestinal conditions (*n* = 348)	108 (31%)	18 (24%)	20 (28%)	23 (30%)	14 (28%)	33 (44%)	0.10 (FE)
Trouble sleeping (*n* = 351)	182 (52%)	74 (21%)	73 (21%)	78 (22%)	50 (14%)	76 (22%)	< 0.001 (FE)
High cholesterol (*n* = 347)	111 (32%)	21 (28%)	20 (27%)	35 (33%)	15 (31%)	30 (40%)	0.50 (FE)
**Living with 2 or more concurrent health conditions in addition to HIV (*n* = 357)**	274 (77%)	52 (69%)	50 (68%)	65 (83%)	33 (66%)	74 (93%)	< 0.001 (FE)
**Cigarette smoking history (last 30 days) (*n* = 349)**							
I currently smoke or I smoke occasionally	102 (29%)	19 (26%)	22 (30%)	13 (17%)	13 (26%)	35 (44%)	0.011 (MC)
I am a former smoker	117 (33%)	27 (37%)	19 (26%)	35 (46%)	14 (28%)	22 (28%)	
I have never been a smoker	130 (37%)	28 (28%)	31 (43%)	29 (38%)	21 (42%)	21 (26%)	
**Substance use in the past 30 days**							
Alcohol (*n* = 351)	196 (56%)	53 (72%)	46 (63%)	37 (47%)	30 (60%)	30 (40%)	0.001 (MC)
Cannabis (*n* = 349)	91 (26%)	10 (14%)	11 (16%)	27 (35%)	7 (14%)	36 (47%)	< 0.001 (MC)
Non-Legalized Substances—Methamphetamines, cocaine, heroin, or non-prescribed opioids (*n* = 353)	54 (15%)	7 (10%)	10 (14%)	9 (12%)	3 (6%)	25 (32%)	0.001 (MC)
**Providers, services or supports accessed in the last 6 months**							
HIV Provider (*n* = 352)	235 (67%)	47 (63%)	48 (66%)	55 (72%)	32 (64%)	53 (68%)	0.751 (FE)
Primary Care Provider (nurse practitioner, doctor, or physician) (*n* = 355)	191 (54%)	31 (41%)	34 (46%)	50 (64%)	19 (38%)	57 (73%)	< 0.001 (MC)
Substance Use Provider (doctor or physician, counsellor, therapist, chem sex support worker or drug support services) (*n* = 354)	36 (10%)	4 (5%)	9 (12%)	4 (5%)	1 (2%)	18 (23%)	0.003 (FE)
Psychiatrist (*n* = 353)	55 (16%)	2 (3%)	11 (15%)	13 (17%)	3 (6%)	26 (33%)	< 0.001 (FE)
Psychologist (*n* = 349)	38 (11%)	2 (3%)	9 (12%)	10 (13%)	4 (8%)	13 (17%)	0.046 (FE)
Nurse (*n* = 347)	77 (22%)	5 (7%)	14 (19%)	19 (25%)	8 (17%)	31 (40%)	< 0.001 (FE)
Occupational Therapist (*n* = 347)	27 (8%)	0 (0%)	5 (7%)	3 (4%)	1 (2%)	18 (23%)	< 0.001 (FE)
Physiotherapist (*n* = 349)	42 (12%)	3 (4%)	5 (7%)	5 (6%)	4 (8%)	25 (32%)	< 0.001 (FE)
Social Worker (*n* = 349)	54 (16%)	1 (1%)	4 (6%)	17 (22%)	1 (2%)	31 (39%)	< 0.001 (FE)
Community-Based Organization (*n* = 321)	70 (22%)	7 (10%)	8 (12%)	12 (18%)	5 (11%)	38 (53%)	< 0.001 (CS)
Family (*n* = 355)	83 (23%)	7 (10%)	17 (23%)	28 (36%)	8 (16%)	23 (29%)	0.001 (FE)
Partner or spouse (*n* = 354)	16 (21%)	16 (21%)	20 (27%)	18 (23%)	15 (31%)	14 (18%)	0.484 (FE)
Friends (*n* = 353)	137 (39%)	21 (28%)	26 (36%)	33 (43%)	12 (25%)	45 (57%)	< 0.001 (FE)
**General health status (*n* = 357)**							
Excellent	50 (14%)	10 (13%)	10 (14%)	12 (15%)	9 (18%)	9 (11%)	0.019 (FE)
Very good	96 (27%)	23 (31%)	17 (23%)	23 (30%)	21 (42%)	12 (15%)	
Good	102 (29%)	24 (32%)	14 (19%)	21 (27%)	12 (24%)	31 (39%)	
Fair	77 (22%)	14 (19%)	21 (28%)	17 (22%)	7 (14%)	18 (23%)	
Poor	32 (9%)	4 (5%)	12 (16%)	1 (2%)	1 (2%)	10 (13%)	
**Engaged in ≥ 150 min of moderate to vigorous aerobic physical activity in the past week (*n* = 338)**	143 (40%)	38 (51%)	28 (38%)	28 (36%)	18 (36%)	31 (39%)	0.29 (CS)
**Current Exercise History (*n* = 355)**							
Do not exercise, do not intend to start	42 (12%)	6 (8%)	11 (15%)	7 (9%)	3 (6%)	15 (19%)	0.018 (MC)
Do not exercise, but thinking of starting	58 (16%)	10 (14%)	8 (11%)	14 (18%)	11 (22%)	15 (19%)	
Exercise but not regularly	90 (25%)	16 (22%)	19 (26%)	16 (21%)	18 (36%)	21 (27%)	
Exercise regularly but only begun so in last 6 months	36 (10%)	7 (10%)	9 (12%)	13 (17%)	0 (0%)	7 (9%)	
Exercise regularly and have done so for > 6 months	101 (29%)	30 (41%)	19 (26%)	21 (27%)	17 (34%)	14 (18%)	
Exercised regularly in past but not doing so currently	28 (8%)	5 (7%)	8 (11%)	7 (9%)	1 (2%)	7 (9%)	
**Days of exercise in past week (*n* = 357)**							
0 (none)	97 (27%)	20 (27%)	22 (30%)	20 (26%)	10 (20%)	25 (31%)	0.86 (MC)
1 day	38 (11%)	8 (11%)	9 (12%)	8 (10%)	3 (6%)	10 (13%)	
2 days	43 (12%)	11 (15%)	4 (5%)	11 (14%)	9 (18%)	8 (10%)	
3 days	46 (13%)	9 (12%)	10 (14%)	11 (14%)	7 (14%)	9 (11%)	
4 days	41 (12%)	12 (16%)	7 (10%)	8 (10%)	5 (10%)	9 (11%)	
5 days	32 (9%)	3 (4%)	9 (12%)	7 (9%)	7 (14%)	6 (8%)	
6 days	21 (6%)	6 (8%)	6 (8%)	5 (6%)	2 (4%)	2 (3%)	
7 days	39 (11%)	6 (8%)	7 (10%)	8 (10%)	7 (14%)	11 (14%)	

Statistical tests: *KW* Kruskal Wallis, *CS* Chi Square, *FE* Fisher’s Exact, *MC* Monte Carlo Simulation

Other^a^ include: Agokwa – male body/changing one’s in the Ojibwa Nation, Bender Gender

### Mode of administration

The mode of administration at the Ireland, UK, and US sites were primarily remote whereby participants completed the questionnaires independently online by accessing the link to the questionnaire via SMS text or email (76%), whereas at the Canadian site, most participants completed the questionnaires in-person using a tablet (24%). There were differences in characteristics based on mode of administration at T1 across cities, given the mode of administration was dependent to each city (see Additional file 5).

### Internal consistency reliability

The EDQ met criteria for internal consistency across domains of the presence, severity and episodic scales (ICC > 0.7). Cronbach’s alpha for EDQ severity scores ranged from 0.84 (social domain) to 0.91 (day domain), for EDQ presence scores ranged from 0.72 (uncertainty domain) to 0.88 (day domain), and for EDQ episodic scores ranged from 0.87 (physical, cognitive, mental-emotional domains) to 0.89 (uncertainty domain) (Table 2). Lower bound CIs for all Cronbach’s alpha were > 0.70, with the exception of the uncertainty domain (0.68) of the EDQ presence scale (Table 2).

**Table 2:** Internal consistency reliability for Episodic Disability Questionnaire (EDQ) Domain scores (*n* = 359 participants)

		**Severity Scale**		**Presence Scale**		**Episodic Scale**	
**EDQ Domain**	**# items**	**Cronbach Alpha**	**95% CI**	**Cronbach Alpha**	**95% CI**	**Cronbach Alpha**	**95% CI**
Physical	10	0.89	0.87, 0.90	0.84	0.82, 0.86	0.87	0.84, 0.89
Cognitive	3	0.85	0.82, 0.88	0.78	0.75, 0.82	0.87	0.84, 0.89
Mental Emotional	5	0.88	0.86, 0.90	0.83	0.80, 0.86	0.87	0.85, 0.89
Uncertainty	5	0.86	0.84, 0.88	0.72	0.68, 0.77	0.89	0.87, 0.90
Day to Day	5	0.91	0.89, 0.92	0.88	0.86, 0.90	0.88	0.87, 0.90
Social	7	0.84	0.82, 0.86	0.79	0.76, 0.83	0.88	0.86, 0.90

*CI* Confidence Interval

> 0.7 considered acceptable

### Test–retest reliability

Overall, the EDQ met criteria for test–retest reliability for EDQ severity domains with ICCs ranging from 0.79 (physical domain) to 0.88 (day domain) and for EDQ presence domains ranging from 0.71 (uncertainty domain) to 0.85 (day domain) (Table 3). Lower bound CIs for all ICCs were > 0.70 with the exception of the physical (0.69) and uncertainty (0.64) domains of the EDQ presence scale (Table 3).

**Table 3:** Test–retest reliability for Episodic Disability Questionnaire (EDQ) Domains of severity and presence scales (*n* = 274)

		**Severity Scale**		**Presence Scale**	
**EDQ Domain**	**# items**	**ICC**	**95% CI**	**ICC**	**95% CI**
Physical	10	0.79	0.71, 0.85	0.76	0.69, 0.82
Cognitive	3	0.84	0.80, 0.87	0.77	0.72, 0.82
Mental Emotional	5	0.84	0.79, 0.87	0.78	0.73, 0.82
Uncertainty	5	0.81	0.71, 0.87	0.71	0.64, 0.77
Day to Day	5	0.88	0.85, 0.91	0.85	0.82, 0.88
Social	7	0.85	0.80, 0.88	0.80	0.75, 0.84

*ICC* Intra Class Correlation Coefficient, *CI* Confidence Interval

For remote independent administration (*n* = 209), the EDQ severity domains, met criteria for test–retest reliability with ICCs ranging from 0.79 (physical, uncertainty domains) to 0.89 (day domain) and for EDQ presence domains ranging from 0.72 (uncertainty) to 0.85 (day domain) (see Additional file 4). For in-person administration (*n* = 24), EDQ severity domains met criteria for test–retest reliability with ICCs ranging from 0.73 (cognitive domain) to 0.87 (social domain) and for two of six EDQ presence domains ranging from 0.48 (uncertainty) to 0.82 (social domain) (see Additional file 6).

### Minimum Detectable Change (MDC)

As shown in Table 4, the severity scale for each domain demonstrated highest precision (MDC95% range: 19–25), followed by the presence (MDC95% range: 37–54) and episodic scales (MDC95% range: 44–76) (Table 4).

**Table 4:** Minimum Detectable Change (MDC) for Episodic Disability Questionnaire (EDQ) Scales (*n* = 274 participants)

	**Severity Scale**		**Presence Scale**		**Episodic Scale**	
**EDQ Domain**	**MDC (90%)**	**MDC (95%)**	**MDC (90%)**	**MDC (95%)**	**MDC (90%)**	**MDC (95%)**
Physical	18	22	31	37	43	51
Cognitive	19	23	45	54	64	76
Mental Emotional	21	25	39	47	56	66
Uncertainty	21	25	38	45	54	65
Day to Day	16	19	36	43	46	55
Social	17	20	31	37	37	44

MDC (90%): Minimum detectable change (MDC) for 90% confidence, MDC (95%): Minimum detectable change (MDC) for 95% confidence

### Construct validity hypotheses

Of the 36 primary hypotheses, 29 (81%) were confirmed; and of the 44 secondary exploratory hypotheses, 36 (82%) were confirmed, supporting construct validity for use with adults living with HIV (see Additional file 7).

## Discussion

The EDQ scales possess internal consistency reliability, and the EDQ severity and presence scales possess construct validity and test–retest reliability with limited precision and when administered electronically among adults living with HIV across eight clinical settings in four countries, Canada, Ireland, United Kingdom and the United States. This work goes beyond past HDQ measurement property assessment that focused on severity scales only [12–16] and SF-HDQ property assessment with the Rasch interval level of measurement that demonstrated structural validity, reliability and sensibility for use among adults living with HIV [17, 18].

The EDQ presence, severity and episodic scales possessed internal consistency reliability with all lower bound CIs of Cronbach’s alphas > 0.70, with the exception of the uncertainty domain in the EDQ presence scale (0.68) suggesting the domains are homogeneous and collectively measure the broader construct of disability. This aligns with earlier internal consistency reliability assessment of severity scales of the SF-HDQ, whereby Cronbach’s alphas ranged from 0.78–0.85 [17] and original HDQ assessment in Canada and Ireland (Cronbach alpha range: 0.81–0.95) [12]. We anticipated lower Cronbach’s alpha in this study, given the EDQ has fewer number of items in each domain (3–10 items) compared with the original long-form HDQ (3–20 items) [12].

Our assessment for test–retest reliability of the EDQ severity and presence scales demonstrated ICCs > 0.70. Lower bound CIs for all ICCs were > 0.70, with the exception of the physical (0.69) and uncertainty (0.64) domains of the EDQ presence scale (Table 3). While community members living with HIV and clinicians highlighted the utility of the precursor SF-HDQ as an individual assessment of disability [18], this requires higher thresholds for reliability (> 0.80) [43], which we did not achieve in this study. Our assessment of ICCs involving only two points in time, may not account for the potential daily fluctuations in disability, which would have influenced the EDQ scores at T2 interpreted as error, hence the ICCs represented may underestimate the test–retest reliability of the EDQ. Future reliability of the EDQ with more repeated measures over time would be beneficial so that the tool may have utility for administration at routine HIV clinical care visits. Results suggest the EDQ may be positioned for group-based or program evaluation purposes rather than assessing disability or its use as an evaluative measure to assess change at an individual level. Furthermore, we acknowledge the difficulty to identify source of measurement error when the mode of EDQ administration was dependent on the recruitment site with a diversity in populations. Ultimately, clinicians must determine what level of error they are willing to tolerate given the EDQ’s intended use and the types of decisions that might be made based on the scores (e.g. referral to services, eligibility for disability income support, etc.) in clinical practice.

For test–retest reliability, it is important to consider potential sources of error such as mode of administration (electronic or paper-based), type of administration (interview administered versus self-completion), or setting [44]. The primary mode of administration in this study was remote whereby participants received a link via SMS or email to independently complete the questionnaire opposed to in-person administration via tablet at the Toronto site. Lower ICCs reported with in-person (tablet) administration may be attributed to the small sample size (*n* = 24) (see Additional file 6). Nevertheless, our provision of multiple options for mode of administration is a strength of the study and highlights the utility of the EDQ across different clinical settings.

The EDQ demonstrated lack of precision across the severity, presence and episodic scales (Table 4). This highlights the limitation for the EDQ to measure change in disability as distinct from day-to-day variability. While further work is needed to determine responsiveness to change in disability over time, results suggest the EDQ may only be able to detect large changes in disability that surpass day to day variability and measurement error.

### Implications for practice and research

The EDQ has potential for use in clinical and community-based settings to describe disability, facilitate communication among providers and patients, facilitate goal setting, and inform allocation of resources for service provision [6, 8, 45–47]. Clinicians and persons living with HIV can use the EDQ to view and interpret the distribution of scores across the domains to specifically indicate what dimensions may pose more (or less) of a challenge.

Our test–retest reliability and construct validity assessment was with the EDQ presence and severity scales only, as we did not expect consistency in a scale that refers to fluctuations in the past week, and the criterion measures did not measure the episodic nature of health challenges. While the episodic scale of the EDQ is an important feature to characterize disability experiences, [16, 18], and is unique to other disability measures [24, 48], the use of the scale should be descriptive in nature to assist in providing a broader picture of the disability experience at a point in time.

This work directly builds on the SF-HDQ and its utility and sensibility with adults living with HIV in community and clinical settings [18]. Clinicians and persons living with HIV may use the EDQ or its precursor SF-HDQ in clinical practice. Both possess the same number of items, structural validity, and scoring algorithm based on the Rasch logit scale [17]. However, we anticipate the EDQ will be of greater use among adults aging with HIV who may experience disability attributed to other concurrent health conditions and not specifically HIV. Furthermore, using the EDQ will enable cross comparisons with other chronic conditions in the future. Current research is assessing the utility and properties of the EDQ with other chronic and episodic conditions [49].

### Limitations

We were unable to determine whether mode of administration or site influenced the EDQ properties as we were unable to disassociate mode of administration from clinical site, country, and participant characteristics. Nevertheless, differences in participant characteristics and EDQ scores, reflected the types of populations served at each site and we consider the heterogeneity of the sample across the sites a strength of our study. Only 11% of the sample were women, which under-represents the proportion of women living with HIV in these countries [50, 51]. Research is currently assessing the measurement properties of the EDQ specifically with women living with HIV in the UK [52]. Finally, our results may not be transferable to adults living with HIV in low or middle-income countries [53].

## Conclusions

The 35-item EDQ is a newly established generic disability patient-reported outcome measure that measures the presence, severity and episodic nature of disability across six domains (physical, cognitive, mental-emotional, daily activities, uncertainty, and challenges to social inclusion). The EDQ scale possess internal consistency reliability, and the EDQ severity and presence scales possess construct validity and test–retest reliability with limited precision when administered electronically with adults living with HIV across eight clinical settings in four countries. Future work may explore the use of the EDQ in other chronic and episodic conditions.

### Supplementary Information

**Additional file 1.** Episodic Disability Questionnaire (EDQ) Items.

**Additional file 2.** Participant Characteristic and EDQ Differences for Participants who Completed T2 EDQ and Did Not Complete T2 EDQ.

**Additional file 3.** Participant Characteristic Differences Across Sites – All Characteristics.

**Additional file 4.** Episodic Disability Questionnaire (EDQ) Scores by City at Time 1 (n=359).

**Additional file 5.** Characteristics of Participants based on Mode of Administration at Time 1 (n=233 participants with similar modes of administration at T1 and T2).

**Additional file 6.** Mode of Administration - Test-Retest Reliability of the Episodic Disability Questionnaire (EDQ) Severity and Presence Scales.

**Additional file 7.** Construct Validity Hypotheses Testing and Results of the Episodic Disability Questionnaire (EDQ).

## Data Availability

The data supporting the conclusions of this article are included within the article and its additional files. The data used and/or analyzed during this study may be available from the corresponding author on reasonable request.

## Abbreviations

CI: Confidence Interval
HIV: Human Immunodeficiency Virus
ICC: Intraclass Correlation Coefficient
PROM: Patient-Reported Outcome Measure
EDQ: Episodic Disability Questionnaire
HDQ: HIV Disability Questionnaire
MDC: Minimum Detectable Change
SF-HDQ: Short-Form HIV Disability Questionnaire
