# Supplementary material for: Episodic disability questionnaire (EDQ) measurement properties among adults living with HIV in Canada, Ireland, United Kingdom, and United States
Source: BMC Infect Dis. 2024 Jan 10;24:71. doi: 10.1186/s12879-023-08958-7 (PMC10782617; doi:10.1186/s12879-023-08958-7)
Supplement: Supplementary file 1 — Additional file 1. Episodic Disability Questionnaire (EDQ) Items. [file 12879_2023_8958_MOESM1_ESM.pdf]

Additional File 1 – Episodic Disability Questionnaire (EDQ) Items

Physical Domain	
**Item #**	**Item**
1	I feel fatigued (or tired) when doing my usual activities.
2	I have headaches.
3	I have aches or pains.
4	I have numbness or tingling in my hands or feet.
5	I have shortness of breath.
6	I feel nauseous.*
7	I have fever, chills, or sweats.
8	I have muscle cramps.
9	I have trouble sleeping.
10	I feel dizzy.
**Physical Total Number of Items**	**10 items**

Cognitive Domain	
**Item #**	**Item**
11	I have trouble remembering things like appointments and when to take my medication.
12	I have trouble thinking clearly.
13	I have trouble concentrating.
**Cognitive Total Number of Items**	**3 items**

Mental-Emotional Domain	
**Item #**	**Item**
14	I feel anxious.
15	I feel sad, down, or depressed.
16	I am afraid for my future.
17	I lack confidence around others.
18	I feel lonely.
**Mental-Emotional Total Number of Items**	**5 items**

Uncertainty or Worry about the Future Domain	
**Item #**	**Item**
19	I worry about my future health.
20	I worry about my lab (blood) or other test results (or investigations).
21	I worry about the side effects of treatments (including medications).
22	I worry about my income or financial security.
23	I worry what might happen to my family and friends if I get sick.
**Uncertainty Total Number of Items**	**5 items**

Day-to-Day Activities Domain	
**Item #**	**Item**
24	I am unsteady on my feet.
25	I have trouble walking or climbing stairs.
26	I have trouble doing household chores such as cleaning, doing dishes, laundry, and cooking.
27	I have trouble taking part in leisure or recreational activities, such as exercise or dancing.
28	I have trouble getting out to do errands, such as grocery shopping, banking, or doctor's appointments
**Day Total Number of Items**	**5 items**

Additional File 1 – Episodic Disability Questionnaire (EDQ) Items

Social Inclusion Domain	
**Item #**	**Item**
29	I find hard to meet the needs of those I care for (such as children, parents, grandparents, partners, or pets). (0=not at all or not applicable)
30	I feel that my work or volunteer performance is limited because of my illness (0=not at all or not applicable).
31	I struggle to maintain safe and stable housing.*
32	I find it hard to ask others for help when go through an episode of illness.
33	I find it hard to start new, intimate, sexual relationships. (0=not at all or not applicable)*
34	I tend to isolate myself from others.
35	Because of my financial situation, I find it hard to take part in leisure or recreational activities such as going to the movies, out to dinner or on vacation.
**Social Inclusion Total Number of Items**	**7 items**

Good Day or Bad Day Question	
**Item #**	**Item**
36	People living with long-term health conditions sometimes talk about having 'good days' and 'bad days'. This refers to the 'ups and downs' or periods of wellness and illness.<br>In terms of your overall health, are you having a good day or a bad day today?

LEGEND: **Presence and Severity Scales*: Response options are 0-4 for all items with the exception of Item #6 (response options 0-3) and Item #31 and #33 (response options 0=2).
Episodic Scale: For each item, respondents are asked, 'Has this challenge fluctuated (or changed) over the past week? Response options: Yes or No.
