# Supplementary material for: Episodic disability questionnaire (EDQ) measurement properties among adults living with HIV in Canada, Ireland, United Kingdom, and United States
Source: BMC Infect Dis. 2024 Jan 10;24:71. doi: 10.1186/s12879-023-08958-7 (PMC10782617; doi:10.1186/s12879-023-08958-7)
Supplement: Supplementary file 2 — Additional file 2. Participant Characteristic and EDQ Differences for Participants who Completed T2 EDQ and Did Not Complete T2 EDQ. [file 12879_2023_8958_MOESM2_ESM.pdf]

Additional file 2 - Participant Characteristic and EDQ Differences for Participants who Completed T2 EDQ and Did Not Complete T2 EDQ

Characteristic	Full Sample (n=359)	Did not Complete T2 (n=38)	Completed T2 (n=321)	p-value	Statistical Test
**Age**				0.29	Kruskal Wallis
Mean (sd)	50.6 (11.8)	48.7 (13.8)	50.9 (11.5)		
Median (Min,Max)	51 (20, 82)	48 (25, 79)	52 (20, 82)		
Missing	6	1	5		
**Gender**				0.83	Fisher Exact
Woman: Cis-Woman	40 (11)	4 (11)	36 (11)		
Man: Cis-Man	293 (83)	32 (86)	261 (82)		
All Other*	21 (6)	1 (3)	20 (6)		
Missing	5	1	4		
**Sex**				0.12	Fisher Exact
Female	40 (11)	4 (11)	36 (11)		
Male	314 (88)	32 (86)	282 (89)		
Intersex	1 (0)	1 (3)	0 (0)		
Missing	4	1	3		
**Relationship status**				0.60	Chi Sq
Single	196 (58)	17 (50)	179 (59)		
Married or common law	88 (26)	10 (29)	78 (26)		
Separated or divorced or widowed	55 (16)	7 (21)	48 (16)		
Missing	20	4	16		
**Have children**				0.85	Chi Sq
Yes	86 (24)	8 (22)	78 (25)		
No	269 (76)	29 (78)	240 (75)		
Missing	4	1	3		
**Live alone**				0.19	Chi Sq
Yes	186 (52)	15 (41)	171 (53)		
No	171 (48)	22 (59)	149 (47)		
Missing	2	0	1		
**Source of Income**				0.14	Fisher Exact
Employment (Full, Part-Time, or Self)	177 (50)	22 (59)	155 (48)		
Income Support (e.g. Disability, Welfare, Worker's Compensation, Employment Insurance or Long Term Disability)	121 (34)	7 (19)	114 (36)		
Pension, Student Loans, or Savings	41 (11)	5 (14)	36 (11)		
Under the table work or Street Related Work (e. g. panhandling)	3 (1)	1 (3)	2 (1)		
Other	15 (4)	2 (5)	13 (4)		
Missing	2	1	1		

Additional file 2 –Participant Characteristic and EDQ Differences for Participants who Completed T2 EDQ and Did Not Complete T2 EDQ

Characteristic	Full Sample (n=359)	Did not Complete T2 (n=38)	Completed T2 (n=321)	p-value	Statistical Test
**Employment status**				0.85	Fisher Exact
Employed (Full time or Part time)	167 (47)	19 (54)	148 (46)		
Student, Retired or Volunteering	59 (17)	5 (14)	54 (17)		
Unemployed or On Disability	124 (35)	11 (31)	113 (35)		
Other	5 (1)	0 (0)	5 (2)		
Missing	4	3	1		
**Highest level of education**				0.38	Chi Sq
No formal education; secondary school completed	88 (25)	7 (19)	81 (25)		
Completed trade or technical training (received certification /diploma) or completed college (received degree or diploma)	132 (37)	12 (32)	120 (38)		
Completed university (received degree) or postgraduate education	137 (38)	18 (49)	119 (37)		
Missing	2	1	1		
**Race White**				0.096	Chi Sq
Yes	255 (81)	22 (69)	233 (83)		
No	59 (19)	10 (31)	49 (17)		
Missing	45	6	39		
**Year of HIV diagnosis**				0.60	Wilcoxon Rank Sum
Mean (sd)	2004 (10)	2006 (8)	2004 (11)		
Median (Min,Max)	2005 (1980, 2021)	2006 (1996, 2020)	2005 (1980, 2021)		
Missing	115	16	99		
**In HIV care**				1.00	Fisher Exact
Yes	339 (95)	35 (95)	304 (95)		
No	18 (5)	2 (5)	16 (5)		
Missing	2	1	1		
**Antiretroviral use**				0.48	Fisher Exact
Yes	351 (98)	36 (97)	315 (98)		
No	6 (2)	1 (3)	5 (2)		
Missing	2	1	1		
**Undetectable viral load**				0.092	Fisher Exact
Yes	328 (93)	35 (100)	293 (92)		
No	26 (7)	0 (0)	26 (8)		
Missing	5	3	2		
**Concurrent health condition – addiction or substance use disorder**				1.00	Chi Sq
No	276 (78)	28 (78)	248 (78)		
Yes	77 (22)	8 (22)	69 (22)		
Missing	6	2	4		
**Concurrent health condition – chronic joint pain**				0.11	Chi Sq
No	233 (67)	29 (81)	204 (66)		
Yes	113 (33)	7 (19)	106 (34)		
Missing	13	2	11		

Additional file 2 –Participant Characteristic and EDQ Differences for Participants who Completed T2 EDQ and Did Not Complete T2 EDQ

Characteristic	Full Sample (n=359)	Did not Complete T2 (n=38)	Completed T2 (n=321)	p-value	Statistical Test
**Concurrent health condition – chronic neuropathic pain**				0.43	Chi Sq
No	266 (77)	30 (83)	236 (76)		
Yes	81 (23)	6 (17)	75 (24)		
Missing	12	2	10		
**Concurrent health condition – chronic soft tissue pain**				0.69	Chi Sq
No	247 (71)	27 (75)	220 (70)		
Yes	102 (29)	9 (25)	93 (30)		
Missing	10	2	8		
**Concurrent health condition – cognitive decline**				0.81	Chi Sq
No	254 (73)	25 (69)	229 (73)		
Yes	96 (27)	11 (31)	85 (27)		
Missing	9	2	7		
**Concurrent health condition – gastrointestinal conditions**				0.16	Chi Sq
No	240 (69)	29 (81)	211 (68)		
Yes	108 (31)	7 (19)	101 (32)		
Missing	11	2	9		
**Concurrent health condition – high blood pressure**				0.094	Chi Sq
No	247 (71)	31 (84)	216 (69)		
Yes	103 (29)	6 (16)	97 (31)		
Missing	9	1	8		
**Concurrent health condition – high cholesterol**				0.047	Chi Sq
No	236 (68)	31 (84)	205 (66)		
Yes	111 (32)	6 (16)	105 (34)		
Missing	12	1	11		
**Concurrent health condition – mental health condition (anxiety, depression)**				0.69	Chi Sq
No	189 (54)	21 (58)	168 (53)		
Yes	162 (46)	15 (42)	147 (47)		
Missing	8	2	6		
**Concurrent health condition – trouble sleeping (insomnia)**				1.00	Chi Sq
No	169 (48)	18 (49)	151 (48)		
Yes	182 (52)	19 (51)	163 (52)		
Missing	8	1	7		
**Smoking history**				0.66	Fisher Exact
I currently smoke (in the last 30 days)	68 (19)	7 (20)	61 (19)		
I smoke occasionally (in the last 30 days)	34 (10)	4 (11)	30 (9)		
I am a former smoker (have not smoked in the last 30 days)	117 (33)	9 (26)	108 (34)		
I have never been a smoker	130 (37)	14 (40)	116 (36)		
I prefer not to answer	5 (1)	1 (3)	4 (1)		
Missing	5	3	2		

Additional file 2 –Participant Characteristic and EDQ Differences for Participants who Completed T2 EDQ and Did Not Complete T2 EDQ

Characteristic	Full Sample (n=359)	Did not Complete T2 (n=38)	Completed T2 (n=321)	p-value	Statistical Test
**General health status**				0.33	Fisher Exact
Excellent	50 (14)	9 (24)	41 (13)		
Very Good	96 (27)	11 (30)	85 (27)		
Good	102 (29)	7 (19)	95 (30)		
Fair	77 (22)	7 (19)	70 (22)		
Poor	32 (9)	3 (8)	29 (9)		
Missing	2	1	1		
**Health status compared to 1 year ago**				0.93	Fisher Exact
Much better now than 1 year ago	54 (15)	6 (16)	48 (15)		
Somewhat better now than 1 year ago	64 (18)	6 (16)	58 (18)		
About the same as 1 year ago	135 (38)	13 (35)	122 (38)		
Somewhat worse now than 1 year ago	76 (21)	8 (22)	68 (21)		
Much worse now than 1 year ago	28 (8)	4 (11)	24 (7)		
Missing	2	1	1		
**Aerobic exercise ($\geq$150 min moderate to vigorous aerobic physical activity in the past week)**				0.15	Fisher Exact
Yes	143 (40)	16 (43)	127 (40)		
No	195 (55)	17 (46)	178 (56)		
I don't know	18 (5)	4 (11)	14 (4)		
Missing	3	1	2		
**Strengthening exercise (at least 2 days in the past week)**				0.89	Fisher Exact
Yes	103 (29)	12 (32)	91 (28)		
No	242 (68)	24 (65)	218 (68)		
I don't know	12 (3)	1 (3)	11 (3)		
Missing	2	1	1		
**Exercise status**				0.17	Fisher Exact
Do not exercise, do not intend to start	42 (12)	3 (8)	39 (12)		
Do not exercise, thinking of starting	58 (16)	8 (22)	50 (16)		
Exercise but not regularly	90 (25)	9 (24)	81 (25)		
Exercise regularly but only begun so in last 6 months	36 (10)	0 (0)	36 (11)		
Exercise regularly and have done so for >6 months.	101 (28)	13 (35)	88 (28)		
Exercised regularly in past but not doing so currently	28 (8)	4 (11)	24 (8)		
Missing	4	1	3		

Additional file 2 –Participant Characteristic and EDQ Differences for Participants who Completed T2 EDQ and Did Not Complete T2 EDQ

Characteristic	Full Sample (n=359)	Did not Complete T2 (n=38)	Completed T2 (n=321)	p-value	Statistical Test
**Number of days exercised (30 min or more) in the past week**				0.54	Fisher Exact
0 (None)	97 (27)	11 (30)	86 (27)		
1 day	38 (11)	3 (8)	35 (11)		
2 days	43 (12)	4 (11)	39 (12)		
3 days	46 (13)	3 (8)	43 (13)		
4 days	41 (11)	3 (8)	38 (12)		
5 days	32 (9)	2 (5)	30 (9)		
6 days	21 (6)	3 (8)	18 (6)		
7 days	39 (11)	8 (22)	31 (10)		
Missing	2	1	1		

Episodic Disability Questionnaire (EDQ) Scores Median Scores (25th, 75th percentile)	Full Sample (n=359)	Did not Complete T2 (n=38)	Completed T2 (n=321)	Rank Sum Test p-value
**Severity Scale**				
Physical	34 (0, 77)	31 (0, 77)	34 (0, 71)	0.24
Cognitive	20 (0, 100)	24 (0, 100)	20 (0, 100)	0.67
Mental-Emotional	41 (0, 100)	46.5 (0.0, 91.0)	41 (0, 100)	0.61
Uncertainty	39 (0, 100)	35 (0, 82)	42 (0, 100)	0.16
Day-to-Day Activities	21 (0, 100)	15 (0, 89)	21 (0, 100)	0.17
Social	34 (0, 75)	35.5 (0.0, 75.0)	34 (0, 75)	0.65
**Presence Scale**				
Physical	52 (0, 100)	45 (0, 100)	52 (0, 100)	0.053
Cognitive	67 (0, 100)	50 (0, 100)	67 (0, 100)	0.86
Mental-Emotional	77 (0, 100)	59 (0, 100)	77 (0, 100)	0.49
Uncertainty	78 (0, 100)	59 (0, 100)	78 (0, 100)	0.013
Day-to-Day Activities	42 (0, 100)	23 (0, 100)	42 (0, 100)	0.091
Social	55 (0, 100)	49.5 (0.0, 100.0)	55 (0, 100)	0.35
**Episodic Scale**				
Physical	10 (0, 100)	10 (0, 100)	10 (0, 100)	0.49
Cognitive	0 (0, 100)	0 (0, 100)	0 (0, 100)	0.64
Mental-Emotional	0 (0, 100)	20 (0, 100)	0 (0, 100)	0.18
Uncertainty	0 (0, 100)	0 (0, 100)	0 (0, 100)	0.45
Day-to-Day Activities	0 (0, 100)	0 (0, 100)	0 (0, 100)	0.29
Social	0 (0, 100)	0 (0, 100)	0 (0, 100)	0.88
