# Supplementary material for: Episodic disability questionnaire (EDQ) measurement properties among adults living with HIV in Canada, Ireland, United Kingdom, and United States
Source: BMC Infect Dis. 2024 Jan 10;24:71. doi: 10.1186/s12879-023-08958-7 (PMC10782617; doi:10.1186/s12879-023-08958-7)
Supplement: Supplementary file 3 — Additional file 3. Participant Characteristic Differences Across Sites – All Characteristics. [file 12879_2023_8958_MOESM3_ESM.pdf]

Additional file 3 - Participant Characteristic Differences Across Sites – All Characteristics

Characteristic	Full Sample (n=359)	Brighton, UK (n=75)	London, UK (n=75)	Denver, United States (n=78)	Dublin, Ireland (n=51)	Toronto, Canada (n=80)	p-value	Statistical Test
**Age**							<0.001	Kruskal Wallis
Mean (sd)	50.6 (11.8)	53.8 (11.3)	50.9 (10.8)	48.9 (12.3)	44.3 (11.3)	53.2 (11.2)		
Median (Min,Max)	51 (20, 82)	54.5 (29.0, 82.0)	51 (31, 79)	50.5 (20.0, 82.0)	43 (28, 71)	55 (25, 71)		
Missing	6	3	1	0	1	1		
**Gender**							<0.001	MC sim
Woman: Cis-Woman	40 (11)	1 (1)	4 (6)	19 (25)	9 (18)	7 (9)		
Man: Cis-Man	293 (83)	74 (99)	65 (90)	52 (68)	41 (82)	61 (76)		
All Other*	21 (6)	0 (0)	3 (4)	6 (8)	0 (0)	12 (15)		
Missing	5	0	3	1	1	0		
**Sex**							<0.001	MC sim
Female	40 (11)	1 (1)	4 (5)	19 (25)	9 (18)	7 (9)		
Male	314 (88)	74 (99)	69 (95)	58 (75)	41 (82)	72 (90)		
Intersex	1 (0)	0 (0)	0 (0)	0 (0)	0 (0)	1 (1)		
Missing	4	0	2	1	1	0		
**Relationship status**							<0.001	Chi Sq
Single	196 (58)	39 (57)	45 (62)	42 (55)	17 (37)	53 (71)		
Married or common law	88 (26)	15 (22)	17 (24)	24 (31)	24 (52)	8 (11)		
Separated or divorced or widowed	55 (16)	15 (22)	10 (14)	11 (14)	5 (11)	14 (19)		
Missing	20	6	3	1	5	5		
**Have children**							0.008	Chi Sq
Yes	86 (24)	13 (17)	10 (14)	28 (36)	12 (24)	23 (29)		
No	269 (76)	62 (83)	64 (86)	49 (64)	38 (76)	56 (71)		
Missing	4	0	1	1	1	1		
**Live alone**							<0.001	Chi Sq
Yes	186 (52)	34 (45)	43 (58)	32 (41)	13 (26)	64 (80)		
No	171 (48)	41 (55)	31 (42)	46 (59)	37 (74)	16 (20)		
Missing	2	0	1	0	1	0		

Additional file 3 –Participant Characteristic Differences Across Cities

Characteristic	Full Sample (n=359)	Brighton, UK (n=75)	London, UK (n=75)	Denver, United States (n=78)	Dublin, Ireland (n=51)	Toronto, Canada (n=80)	p-value	Statistical Test
**Source of Income**							<0.001	MC sim
Employment (Full, Part-Time, or Self)	177 (50)	50 (67)	39 (53)	43 (55)	41 (82)	4 (5)		
Income Support (e.g. Disability, Welfare, Worker's Compensation, Employment Insurance or Long Term Disability)	121 (34)	10 (13)	24 (32)	25 (32)	7 (14)	55 (69)		
Pension, Student Loans, or Savings	41 (11)	13 (17)	6 (8)	3 (4)	1 (2)	18 (22)		
Under the table work or Street Related Work (e. g. panhandling)	3 (1)	0 (0)	0 (0)	1 (1)	0 (0)	2 (3)		
Other	15 (4)	2 (3)	5 (7)	6 (8)	1 (2)	1 (1)		
Missing	2	0	1	0	1	0		
**Employment status**							<0.001	MC sim
Employed (Full time or Part time)	167 (47)	48 (65)	35 (47)	44 (56)	37 (76)	3 (4)		
Student, Retired or Volunteering	59 (17)	15 (20)	11 (15)	6 (8)	5 (10)	22 (28)		
Unemployed or On Disability	124 (35)	11 (15)	26 (35)	26 (33)	6 (12)	55 (69)		
Other	5 (1)	0 (0)	2 (3)	2 (3)	1 (2)	0 (0)		
Missing	4	1	1	0	2	0		
**Highest level of education**							0.29	Chi Sq
No formal education; secondary school completed	88 (25)	15 (20)	18 (24)	19 (24)	11 (22)	25 (31)		
Completed trade or technical training (received certification /diploma) or completed college (received degree or diploma)	132 (37)	27 (36)	24 (32)	30 (38)	16 (32)	35 (44)		
Completed university (received degree) or postgraduate education	137 (38)	33 (44)	32 (43)	29 (37)	23 (46)	20 (25)		
Missing	2	0	1	0	1	0		
**Race White**							0.076	Chi Sq
Yes	255 (81)	64 (86)	53 (90)	52 (72)	36 (80)	50 (78)		
No	59 (19)	10 (14)	6 (10)	20 (28)	9 (20)	14 (22)		
Missing	45	1	16	6	6	16		
**Year of HIV diagnosis**							<0.001	Kruskal Wallis
Mean (sd)	2004 (10)	2005 (9)	2004 (10)	2002 (11)	2013 (6)	2000 (10)		
Median (Min,Max)	2005 (1980, 2021)	2007 (1985, 2021)	2002 (1986, 2021)	2002 (1984, 2021)	2014 (1997, 2020)	2000 (1980, 2019)		
Missing	115	24	25	23	17	26		

Additional file 3 –Participant Characteristic Differences Across Cities

Characteristic	Full Sample (n=359)	Brighton, UK (n=75)	London, UK (n=75)	Denver, United States (n=78)	Dublin, Ireland (n=51)	Toronto, Canada (n=80)	p-value	Statistical Test
**In HIV care**							<0.001	Fisher Exact
Yes	339 (95)	68 (91)	72 (97)	78 (100)	50 (100)	71 (89)		
No	18 (5)	7 (9)	2 (3)	0 (0)	0 (0)	9 (11)		
Missing	2	0	1	0	1	0		
**Antiretroviral use**							0.16	Fisher Exact
Yes	351 (98)	75 (100)	73 (99)	77 (99)	50 (100)	76 (95)		
No	6 (2)	0 (0)	1 (1)	1 (1)	0 (0)	4 (5)		
Missing	2	0	1	0	1	0		
**Undetectable viral load**							0.008	Fisher Exact
Yes	328 (93)	73 (99)	69 (93)	66 (85)	48 (98)	72 (91)		
No	26 (7)	1 (1)	5 (7)	12 (15)	1 (2)	7 (9)		
Missing	5	1	1	0	2	1		
**Concurrent health condition – addiction or substance use disorder**							<0.001	Chi Sq
No	276 (78)	63 (85)	63 (86)	63 (81)	45 (90)	42 (54)		
Yes	77 (22)	11 (15)	10 (14)	15 (19)	5 (10)	36 (46)		
Missing	6	1	2	0	1	2		
**Concurrent health condition – chronic joint pain**							<0.001	MC sim
No	233 (67)	59 (82)	48 (68)	48 (62)	46 (92)	32 (42)		
Yes	113 (33)	13 (18)	23 (32)	29 (38)	4 (8)	44 (58)		
Missing	13	3	4	1	1	4		
**Concurrent health condition – chronic neuropathic pain**							<0.001	MC sim
No	266 (77)	65 (89)	50 (69)	60 (78)	48 (96)	43 (57)		
Yes	81 (23)	8 (11)	22 (31)	17 (22)	2 (4)	32 (43)		
Missing	12	2	3	1	1	5		
**Concurrent health condition – chronic soft tissue pain**							<0.001	Chi Sq
No	247 (71)	63 (85)	52 (72)	54 (70)	43 (86)	35 (46)		
Yes	102 (29)	11 (15)	20 (28)	23 (30)	7 (14)	41 (54)		
Missing	10	1	3	1	1	4		
**Concurrent health condition – cognitive decline**							<0.001	Fisher Exact
No	254 (73)	62 (84)	49 (68)	52 (67)	46 (92)	45 (59)		
Yes	96 (27)	12 (16)	23 (32)	26 (33)	4 (8)	31 (41)		
Missing	9	1	3	0	1	4		

Additional file 3 –Participant Characteristic Differences Across Cities

Characteristic	Full Sample (n=359)	Brighton, UK (n=75)	London, UK (n=75)	Denver, United States (n=78)	Dublin, Ireland (n=51)	Toronto, Canada (n=80)	p-value	Statistical Test
**Concurrent health condition – gastrointestinal conditions**							0.089	Chi Sq
No	240 (69)	56 (76)	52 (72)	54 (70)	36 (72)	42 (56)		
Yes	108 (31)	18 (24)	20 (28)	23 (30)	14 (28)	33 (44)		
Missing	11	1	3	1	1	5		
**Concurrent health condition – high blood pressure**							0.006	Chi Sq
No	247 (71)	59 (80)	53 (73)	46 (60)	42 (84)	47 (62)		
Yes	103 (29)	15 (20)	20 (27)	31 (40)	8 (16)	29 (38)		
Missing	9	1	2	1	1	4		
**Concurrent health condition – high cholesterol**							0.49	Chi Sq
No	236 (68)	53 (72)	53 (73)	51 (67)	34 (69)	45 (60)		
Yes	111 (32)	21 (28)	20 (27)	25 (33)	15 (31)	30 (40)		
Missing	12	1	2	2	2	5		
**Concurrent health condition – mental health condition (anxiety, depression)**							<0.001	Chi Sq
No	189 (54)	52 (70)	39 (53)	41 (53)	33 (67)	24 (31)		
Yes	162 (46)	22 (30)	34 (47)	36 (47)	16 (33)	54 (69)		
Missing	8	1	2	1	2	2		
**Concurrent health condition – trouble sleeping (insomnia)**							<0.001	Chi Sq
No	169 (48)	42 (57)	31 (42)	41 (53)	32 (64)	23 (30)		
Yes	182 (52)	32 (43)	42 (58)	37 (47)	18 (36)	53 (70)		
Missing	8	1	2	0	1	4		
**Smoking history**							0.001	MC sim
I currently smoke (in the last 30 days)	68 (19)	13 (18)	12 (16)	8 (10)	5 (10)	30 (38)		
I smoke occasionally (in the last 30 days)	34 (10)	6 (8)	10 (14)	5 (6)	8 (16)	5 (6)		
I am a former smoker (have not smoked in the last 30 days)	117 (33)	27 (36)	19 (26)	35 (45)	14 (28)	22 (28)		
I have never been a smoker	130 (37)	28 (38)	31 (42)	29 (38)	21 (42)	21 (26)		
I prefer not to answer	5 (1)	0 (0)	1 (1)	0 (0)	2 (4)	2 (3)		
Missing	5	1	2	1	1	0		

Additional file 3 –Participant Characteristic Differences Across Cities

Characteristic	Full Sample (n=359)	Brighton, UK (n=75)	London, UK (n=75)	Denver, United States (n=78)	Dublin, Ireland (n=51)	Toronto, Canada (n=80)	p-value	Statistical Test
**General health status**							0.021	MC sim
Excellent	50 (14)	10 (13)	10 (14)	12 (15)	9 (18)	9 (11)		
Very Good	96 (27)	23 (31)	17 (23)	23 (29)	21 (42)	12 (15)		
Good	102 (29)	24 (32)	14 (19)	21 (27)	12 (24)	31 (39)		
Fair	77 (22)	14 (19)	21 (28)	17 (22)	7 (14)	18 (22)		
Poor	32 (9)	4 (5)	12 (16)	5 (6)	1 (2)	10 (12)		
Missing	2	0	1	0	1	0		
**Health status compared to 1 year ago**							0.011	MC sim
Much better now than 1 year ago	54 (15)	7 (9)	10 (14)	18 (23)	10 (20)	9 (11)		
Somewhat better now than 1 year ago	64 (18)	11 (15)	10 (14)	13 (17)	6 (12)	24 (30)		
About the same as 1 year ago	135 (38)	38 (51)	30 (41)	20 (26)	23 (46)	24 (30)		
Somewhat worse now than 1 year ago	76 (21)	17 (23)	17 (23)	21 (27)	8 (16)	13 (16)		
Much worse now than 1 year ago	28 (8)	2 (3)	7 (9)	6 (8)	3 (6)	10 (12)		
Missing	2	0	1	0	1	0		
**Aerobic exercise (≥150 min moderate to vigorous aerobic physical activity in the past week)**							0.31	MC sim
Yes	143 (40)	38 (51)	28 (38)	28 (36)	18 (36)	31 (39)		
No	195 (55)	33 (44)	45 (61)	46 (60)	28 (56)	43 (54)		
I don't know	18 (5)	4 (5)	1 (1)	3 (4)	4 (8)	6 (7)		
Missing	3	0	1	1	1	0		
**Strengthening exercise (at least 2 days in the past week)**							0.19	MC sim
Yes	103 (29)	23 (31)	22 (30)	23 (29)	9 (18)	26 (32)		
No	242 (68)	52 (69)	49 (66)	53 (68)	40 (80)	48 (60)		
I don't know	12 (3)	0 (0)	3 (4)	2 (3)	1 (2)	6 (7)		
Missing	2	0	1	0	1	0		
**Exercise status**							0.014	MC sim
Do not exercise, do not intend to start	42 (12)	6 (8)	11 (15)	7 (9)	3 (6)	15 (19)		
Do not exercise, thinking of starting	58 (16)	10 (14)	8 (11)	14 (18)	11 (22)	15 (19)		
Exercise but not regularly	90 (25)	16 (22)	19 (26)	16 (21)	18 (36)	21 (27)		
Exercise regularly but only begun so in last 6 months	36 (10)	7 (9)	9 (12)	13 (17)	0 (0)	7 (9)		
Exercise regularly and have done so for >6 months.	101 (28)	30 (41)	19 (26)	21 (27)	17 (34)	14 (18)		
Exercised regularly in past but not doing so currently	28 (8)	5 (7)	8 (11)	7 (9)	1 (2)	7 (9)		
Missing	4	1	1	0	1	1		

Additional file 3 –Participant Characteristic Differences Across Cities

Characteristic	Full Sample (n=359)	Brighton, UK (n=75)	London, UK (n=75)	Denver, United States (n=78)	Dublin, Ireland (n=51)	Toronto, Canada (n=80)	p-value	Statistical Test
**Number of days exercised (30 min or more) in the past week**							0.85	MC sim
0 (None)	97 (27)	20 (27)	22 (30)	20 (26)	10 (20)	25 (31)		
1 day	38 (11)	8 (11)	9 (12)	8 (10)	3 (6)	10 (12)		
2 days	43 (12)	11 (15)	4 (5)	11 (14)	9 (18)	8 (10)		
3 days	46 (13)	9 (12)	10 (14)	11 (14)	7 (14)	9 (11)		
4 days	41 (11)	12 (16)	7 (9)	8 (10)	5 (10)	9 (11)		
5 days	32 (9)	3 (4)	9 (12)	7 (9)	7 (14)	6 (7)		
6 days	21 (6)	6 (8)	6 (8)	5 (6)	2 (4)	2 (3)		
7 days	39 (11)	6 (8)	7 (9)	8 (10)	7 (14)	11 (14)		
Missing	2	0	1	0	1	0		

Statistical tests: KW Kruskal Wallis, CS Chi Square, FE Fisher's Exact, MC, Monte Carlo Simulation;

Other* include: Agokwa – male body/changing one's in the Ojibwa Nation, Bender Gender
