# Supplementary material for: Episodic disability questionnaire (EDQ) measurement properties among adults living with HIV in Canada, Ireland, United Kingdom, and United States
Source: BMC Infect Dis. 2024 Jan 10;24:71. doi: 10.1186/s12879-023-08958-7 (PMC10782617; doi:10.1186/s12879-023-08958-7)
Supplement: Supplementary file 4 — Additional file 4. Episodic Disability Questionnaire (EDQ) Scores by City at Time 1 (n=359). [file 12879_2023_8958_MOESM4_ESM.pdf]

Additional file 4 - Episodic Disability Questionnaire (EDQ) Scores and Criterion Measure Scores by City at Time 1 (n=359)

Additional file 4 - Episodic Disability Questionnaire (EDQ) Scores by City at Time 1 (n=359)

EDQ Scores	Entire Sample (n=359)	Brighton, United Kingdom (n=75)	London, United Kingdom (n=75)	Denver, United States (n=78)	Dublin, Ireland (n=51)	Toronto, Canada (n=80)	Kruskal Wallis Test p-value
**Presence Domain Scores**							
**Physical**							<0.001
Median (25th,75th percentile)	52 (37,75)	45 (37,59)	52 (27,75)	59 (37,75)	45 (15,59)	66 (52,86)	
Min - max	0 – 100	0 – 100	0 – 100	0 – 100	0 - 86	0 – 100	
**Cognitive**							<0.001
Median (25th,75th percentile)	67 (33,100)	67 (0,100)	67 (0,100)	67 (25,100)	33 (0,67)	100 (67,100)	
Min - max	0 – 100	0 – 100	0 – 100	0 – 100	0 – 100	0 – 100	
**Mental-Emotional**							<0.001
Median (25th,75th percentile)	77 (41,100)	41 (22,100)	77 (41,100)	77 (36,100)	59 (41,100)	100 (77,100)	
Min – max	0 - 100	0 – 100	0 – 100	0 – 100	0 – 100	0 – 100	
**Uncertainty**							0.003
Median (25th,75th percentile)	78 (59,100)	78 (41,78)	59 (41,100)	78 (59,100)	78 (41,100)	78 (76,100)	
Min – max	0 - 100	0 – 100	0 – 100	0 – 100	0 – 100	0 – 100	
**Day-to-Day Activities**							<0.001
Median (25th,75th percentile)	42 (0,100)	23 (0,59)	42 (0,100)	42 (0,100)	0 (0,23)	78 (59,100)	
Min – max	0 - 100	0 – 100	0 – 100	0 – 100	0 – 100	0 – 100	
**Social Inclusion**							<0.001
Median (25th,75th percentile)	55 (32,67)	44 (18,67)	55 (32,67)	55 (32,82)	32 (18,55)	67 (55,82)	
Min - max	0 - 100	0 – 100	0 – 100	0 – 100	0 – 100	0 – 100	
**Severity Domain Scores**							
**Physical**							<0.001
Median (25th, 75th percentile)	34 (20,47)	24 (16,43)	31 (16,50)	34 (16,48)	28 (6,39)	45 (31,52)	
Min – max	0 – 77	0 – 53	0 -77	0 - 60	0 – 51	0 – 71	
**Cognitive**							<0.001
Median (25th, 75th percentile)	20 (11, 42)	20 (0,28)	20 (0,48)	24 (8,42)	11 (0,28)	35 (20,48)	
Min - max	0 – 100	0 – 68	0 – 100	0 – 77	0 – 61	0 – 100	
**Mental-Emotional**							<0.001
Median (25th, 75th percentile)	41 (18,53)	26 (10,45)	45 (18,56)	37 (18,53)	32 (18,53)	51 (37,63)	
Min – max	0 - 100	0 – 78	0 – 100	0 – 84	0 – 84	0 - 100	
**Uncertainty**							<0.001
Median (25th, 75th percentile)	39 (30,52)	35 (17,42)	35 (24,55)	42 (30,55)	39 (30,47)	47 (39,57)	
Min – max	0 – 100	0 – 75	0 – 100	0 – 90	0 – 100	0 – 100	
**Day-to-Day Activities**							<0.001
Median (25th, 75th percentile)	21 (0,36)	8 (0,26)	21 (0,42)	21 (0,38)	0 (0,8)	36 (21,44)	
Min – max	0 – 100	0 – 89	0 – 64	0 – 100	0 – 50	0 – 89	
**Social Inclusion**							<0.001
Median (25th, 75th percentile)	34 (15,46)	21 (8,39)	34 (15,44)	37 (15,46)	21 (8,37)	44 (32, 51)	
Min – max	0 – 75	0 - 75	0 – 69	0 – 69	0 – 75	0 – 69	

Additional file 4 - Episodic Disability Questionnaire (EDQ) Scores and Criterion Measure Scores by City at Time 1 (n=359)

EDQ Scores	Entire Sample (n=359)	Brighton, United Kingdom (n=75)	London, United Kingdom (n=75)	Denver, United States (n=78)	Dublin, Ireland (n=51)	Toronto, Canada (n=80)	p-value
**Episodic (fluctuating in the past week) Domain Scores**							
**Physical**<br>Median (25th, 75th percentile)<br>Min – max	10 (0,40)<br>0 – 100	10 (0,40)<br>0 – 90	20 (10,50)<br>0 – 100	10 (0,40)<br>0 – 100	0 (0,20)<br>0 – 80	20 (0,50)<br>0 – 100	<0.001
**Cognitive**<br>Median (25th, 75th percentile)<br>Min – max	0 (0,33)<br>0 – 100	0 (0,0)<br>0 – 100	0 (0,67)<br>0 – 100	0 (0,33)<br>0 – 100	0 (0,0)<br>0 – 100	0 (0,67)<br>0 – 100	<0.001
**Mental-Emotional**<br>Median (25th, 75th percentile)<br>Min – max	0 (0,40)<br>0 – 100	0 (0,20)<br>0 – 100	20 (0,60)<br>0 – 100	0 (0,40)<br>0 – 100	0 (0,20)<br>0 – 100	20 (0,75)<br>0 – 100	0.004
**Uncertainty**<br>Median (25th, 75th percentile)<br>Min – max	0 (0,20)<br>0 – 100	0 (0,0)<br>0 – 100	0 (0,20)<br>0 – 100	0 (0,20)<br>0 – 100	0 (0,0)<br>0 – 100	0 (0,60)<br>0 – 100	0.008
**Day-to-Day Activities**<br>Median (25th, 75th percentile)<br>Min – max	0 (0,20)<br>0 – 100	0 (0,0)<br>0 – 100	0 (0,20)<br>0 – 100	0 (0,20)<br>0 – 100	0 (0,0)<br>0 – 40	0 (0,55)<br>0 – 100	<0.001
**Social Inclusion**<br>Median (25th, 75th percentile)<br>Min - max	0 (0,14)<br>0 – 100	0 (0,0)<br>0 – 100	0 (0,0)<br>0 – 100	0 (0,14)<br>0 – 100	0 (0,0)<br>0 – 100	0 (0,43)<br>0 – 100	<0.001

Additional file 4B –Criterion Measure Scores by City at Time 1 (n=359)

Criterion Measure Scores	Entire Sample (n=359)	Brighton, United Kingdom (n=75)	London, United Kingdom (n=75)	Denver, United States (n=78)	Dublin, Ireland (n=51)	Toronto, Canada (n=80)	Kruskal Wallis Test p-value
**World Health Organization Disability Assessment Schedule (WHODAS 2.0) 36-item (score range: 0-100) (n=358)**							
Median<br>(25th,75th percentile)	23.6<br>(6.8, 42.5)	15.1<br>(3.8, 31.6)	26.1<br>(7.8, 49.2)	21.2<br>(7.6, 41.0)	9.4<br>(3.8, 20.8)	39.1<br>(29.2, 50.9)	<0.001
**Patient Health Questionnaire (PHQ) 8-item (score range: 0-24) (n=358)**							
Median<br>(25th,75th percentile)	6<br>(2, 13)	5.0<br>(1.0, 8.5)	7.0<br>(3.0, 13.8)	5.5<br>(2.0, 11.0)	3.0<br>(1.0, 6.5)	10.5<br>(5.0, 18.0)	<0.001
**Social Support Survey Questionnaire (score range: 0-100) (n=357)**							
Median<br>(25th,75th percentile)	50.0<br>(27.6, 81.6)	64.5<br>(33.6, 89.5)	46.1<br>(30.6, 82.6)	59.2<br>(37.5, 81.2)	52.0<br>(34.2, 87.5)	32.2<br>(16.8, 60.9)	<0.001
**General Health Status Self Reported – Recorded as an Ordinal Variable ranging from 1 (Poor) to 5 (Excellent) (n=357)**							
Median<br>(25th,75th percentile)	3<br>(2, 4)	3<br>(2, 3)	3<br>(2, 4)	3<br>(2, 4)	2<br>(2, 3)	3<br>(2, 4)	0.003

Date Last Revised: December 14, 2023
