# Supplementary material for: Episodic disability questionnaire (EDQ) measurement properties among adults living with HIV in Canada, Ireland, United Kingdom, and United States
Source: BMC Infect Dis. 2024 Jan 10;24:71. doi: 10.1186/s12879-023-08958-7 (PMC10782617; doi:10.1186/s12879-023-08958-7)
Supplement: Supplementary file 5 — Additional file 5. Characteristics of Participants based on Mode of Administration at Time 1 (n=233 participants with similar modes of administration at T1 and T2). [file 12879_2023_8958_MOESM5_ESM.pdf]

Additional file 5 – Characteristics of Participants based on Mode of Administration at Time 1 (n=233 participants with similar modes of administration at T1 and T2)

Characteristic	Full Sample (n=233)	Remote Independent (SMS or email) (n=209)	In person (n=24)	p-value (Statistical Test)
**Age** (n=230) Median (Min, Max)	51 (20, 82)	51 (20, 82)	53 (33, 67)	0.44 (WRS)
**Gender** (n=232)	**# (%)**	**# (%)**	**# (%)**	
Woman: Cis-Woman	26 (11%)	23 (11%)	3 (12%)	<0.001* (FE)
Man: Cis-Man	195 (84%)	180 (87%)	15 (62%)	
Trans Woman: Man to woman	2 (1%)	0 (0%)	2 (8%)	
Two-spirited	5 (2%)	2 (1%)	3 (12%)	
Non-binary	4 (2%)	3 (1%)	1 (4%)	
Missing	1	1	0	
**Relationship Status** (n=225)	**# (%)**	**# (%)**	**# (%)**	
Single	128 (57%)	110 (55%)	18 (75%)	0.06 (FE)
Married or common law	62 (28%)	60 (30%)	2 (8%)	
Separated, divorced, or widowed	35 (16%)	31 (15%)	4 (17%)	
Missing	8	8	0	
**Have children** (n=231)	**# (%)**	**# (%)**	**# (%)**	
Yes	53 (23%)	47 (23%)	6 (26%)	0.79 (FE)
No	178 (77%)	161 (77%)	17 (74%)	
Missing	2	1	1	
**Lives Alone** (n=233)	**# (%)**	**# (%)**	**# (%)**	
Yes	121 (52%)	104 (50%)	17 (71%)	0.08 (CS)
No	112 (48%)	105 (50%)	7 (29%)	
**Source of Income** (n=233)	**# (%)**	**# (%)**	**# (%)**	
Employment (full-time, part-time, or self)	126 (54%)	126 (60%)	0 (0%)	<0.001* (FE)
Income Support (e.g. Disability, Welfare, Worker's Compensation, Employment Insurance or Long Term Disability)	73 (31%)	52 (25%)	21 (88%)	
Pension, Student Loans, or Savings	25 (11%)	22 (11%)	3 (12%)	
Under the table work or Street Related Work (e g panhandling)	1 (0%)	1 (0%)	0 (0%)	
Other	8 (3%)	8 (4%)	0 (0%)	
**Employment Status** (n=233)	**# (%)**	**# (%)**	**# (%)**	
Employment (full-time or part-time)	123 (53%)	123 (59%)	0 (0%)	<0.001* (FE)
Student, Retired or Volunteering	36 (15%)	32 (15%)	4 (17%)	
Unemployed or On Disability	71 (30%)	51 (24%)	20 (83%)	
Other	3 (1%)	3 (1%)	0 (0%)	
**Highest Level of Education** (n=233)	**# (%)**	**# (%)**	**# (%)**	
No formal education; secondary school completed	56 (24%)	47 (22%)	9 (38%)	0.01* (FE)
Completed trade or technical training (received certification /diploma) or completed college (received degree or diploma)	88 (38%)	76 (36%)	12 (50%)	
Completed university (received degree) or postgraduate education	89 (38%)	86 (41%)	3 (12%)	

Additional file 5 – Characteristics of Participants based on Mode of Administration at Time 1 (n=233 participants with similar modes of administration at T1 and T2)

**Characteristic**	**Full Sample (n=233)**	**Remote Independent (SMS or email) (n=209)**	**In person (n=24)**	**p-value (Statistical Test)**
**Race**	**# (%)**	**# (%)**	**# (%)**	
White (n=222)	174 (82%)	160 (82%)	14 (82%)	1.0 (FE)
Black or African American (n=183)	13 (7%)	10 (6)	3 (25%)	0.04* (FE)
Asian (n=180) (origins in far east, south east Asia, or Indian subcontinent including e.g. Cambodia, China, India, Japan, Korea, Malaysia, Pakistan, Philippine Islands, Thailand, Vietnam)	14 (8%)	12 (7%)	2 (18%)	0.21 (FE)
First Nation (Indigenous), Inuit, Metis (n=179)	18 (10%)	17 (10%)	1 (10%)	1.0 (FE)
**Year of HIV Diagnosis** (n=161) Median (Min, Max)	2006 (1980, 2021)	2007 (1980, 2021)	2001 (1983, 2019)	0.33 (WRS)
**Most Recent CD4 Count** (n=231)	**# (%)**	**# (%)**	**# (%)**	
≤200 cells/mm3	27 (12%)	24 (12%)	3 (12%)	0.07 (FE)
201-499 cells/mm3	42 (18%)	36 (17%)	6 (25%)	
≥ 500 cells/mm3	91 (39%)	87 (42%)	4 (17%)	
Don't know	71 (31%)	60 (29%)	11 (46%)	
**Currently taking Antiretroviral Medications** (n=233)	229 (98%)	208 (100%)	21 (88%)	0.004* (FE)
**Missed at least one dose of Antiretroviral Medication in the last 7 days** (n=228)	40 (18%)	34 (16%)	6 (29%)	0.22 (FE)
**Undetectable Viral Load (<50 copies/ml)** (n=232)	212 (91%)	194 (93%)	18 (75%)	0.009* (FE)

*indicates statistical significance p<0.05;

Statistical tests: KW Kruskal Wallis, CS Chi Square, FE Fisher's Exact; WRS Wilcoxon Rank Sum
