# Supplementary material for: Episodic disability questionnaire (EDQ) measurement properties among adults living with HIV in Canada, Ireland, United Kingdom, and United States
Source: BMC Infect Dis. 2024 Jan 10;24:71. doi: 10.1186/s12879-023-08958-7 (PMC10782617; doi:10.1186/s12879-023-08958-7)
Supplement: Supplementary file 6 — Additional file 6. Mode of Administration - Test-Retest Reliability of the Episodic Disability Questionnaire (EDQ) Severity and Presence Scales. [file 12879_2023_8958_MOESM6_ESM.pdf]

Additional file 6 – Mode of Administration - Test-Retest Reliability of the Episodic Disability Questionnaire (EDQ) Severity and Presence Scales

**Remote Independent Administration (SMS or email) (n=209 participants)**				
		**Severity Scale**		**Presence Scale**
**Domain**	**ICC**	**95% CI**	**ICC**	**95% CI**
Physical	0.79	0.72,0.84	0.78	0.71,0.82
Cognitive	0.88	0.85,0.90	0.80	0.75,0.84
Mental Emotional	0.84	0.80,0.88	0.79	0.74,0.83
Uncertainty	0.79	0.68,0.86	0.72	0.64,0.78
Daily	0.89	0.86,0.91	0.85	0.81,0.88
Social	0.83	0.79,0.87	0.78	0.73,0.82
**In-Person Administration (tablet) (n=24 participants)**				
		**Severity Scale**		**Presence Scale**
**Domain**	**ICC**	**95% CI**	**ICC**	**95% CI**
Physical	0.74	0.50,0.87	0.68	0.45,0.83
Cognitive	0.73	0.52,0.86	0.56	0.29,0.76
Mental Emotional	0.76	0.57,0.87	0.65	0.41,0.81
Uncertainty	0.82	0.66,0.91	0.48	0.17,0.70
Daily	0.81	0.66,0.90	0.72	0.50,0.85
Social	0.87	0.76,0.93	0.82	0.67,0.91

ICC: Intraclass Correlation Coefficient; CI: Confidence Interval.
n=233 with similar modes of administration at T1 and T2
ICCs for the Episodic Scale not reported here; all were <0.7 (not shown). We did not expect test-retest reliability in episodic scale given asking about fluctuating in the past week.
