# Supplementary material for: Episodic disability questionnaire (EDQ) measurement properties among adults living with HIV in Canada, Ireland, United Kingdom, and United States
Source: BMC Infect Dis. 2024 Jan 10;24:71. doi: 10.1186/s12879-023-08958-7 (PMC10782617; doi:10.1186/s12879-023-08958-7)
Supplement: Supplementary file 7 — Additional file 7. Construct Validity Hypotheses Testing and Results of the Episodic Disability Questionnaire (EDQ). [file 12879_2023_8958_MOESM7_ESM.pdf]

Additional file 7: Construct Validity Hypotheses Testing and Results of the Episodic Disability Questionnaire (EDQ)

Testing validity hypothesis at Time 1 only for EDQ presence and severity scores (Rasch logit scale). We interpreted lower and upper bound confidence intervals in combination with the correlation coefficient when interpreting hypotheses.

*Indicate primary hypotheses; LB (lower bound of confidence interval); UB (upper bound of confidence interval); All p-values have had a Holm's correction applied to control for multiple comparisons.

Primary Hypotheses (n=36)

Hypothesis 1-4*
Total WHODAS scores (WDAS_SS) will **moderately correlate (≥0.50)** with EDQ SEVERITY domain scores: COGNITION, MENTAL-EMOTIONAL, DIFFICULTIES WITH DAY-TO-DAY ACTIVIITES (DAILY) and SOCIAL

EDQ Domain	N	Spearman Correlation	LB	UB	p-value	Hypothesis confirmed? Yes /No
Severity Cognitive	358	0.74	0.69	0.79	<0.001	Yes
Severity Mental Emotional	358	0.73	0.68	0.78	<0.001	Yes
Severity Daily	358	0.81	0.77	0.84	<0.001	No
Severity Social	358	0.86	0.83	0.88	<0.001	No

Hypothesis 5-6*
Total WHODAS scores (WDAS_SS) will **moderately correlate (≥0.50)** with EDQ SEVERITY domain scores: PHYSICAL and UNCERTAINTY

EDQ Domain	N	Spearman Correlation	LB	UB	p-value	Hypothesis confirmed? Yes /No
Severity Physical	358	0.77	0.73	0.81	<0.001	No
Severity Uncertainty	358	0.69	0.63	0.74	<0.001	Yes

Hypothesis 7-8*
Cognition WHODAS summary scores (WDASDo1) will **strongly correlate (≥0.70**) with the COGNITIVE domain scores of the EDQ (presence and severity)

EDQ Domain	N	Spearman Correlation	LB	UB	p-value	Hypothesis confirmed? Yes /No
Severity Cognitive	359	0.84	0.81	0.87	<0.001	Yes
Presence Cognitive	359	0.79	0.74	0.82	<0.001	Yes

Hypothesis 9-10*
Cognition WHODAS summary scores (WDASDo1) will **moderately correlate (≥0.50)** with the MENTAL-EMOTIONAL domain scores of the EDQ (presence and severity)

EDQ Domain	N	Spearman Correlation	LB	UB	p-value	Hypothesis confirmed? Yes /No
Severity Mental-Emotional	359	0.69	0.63	0.74	<0.001	Yes
Presence Mental-Emotional	359	0.6	0.52	0.66	<0.001	Yes

Hypothesis 11-12*
Mobility WHODAS summary scores will **strongly correlate (>0.70)** with the DIFFICULTIES WITH DAY-TO-DAY ACTIVITIES (Daily) domain scores of the EDQ (presence and severity).

EDQ Domain	N	Spearman Correlation	LB	UB	p-value	Hypothesis confirmed? Yes /No
Severity Daily	359	0.85	0.82	0.88	<0.001	Yes
Presence Daily	359	0.84	0.8	0.87	<0.001	Yes

Hypothesis 13-14*
Self-Care WHODAS summary scores will **strongly correlate (>0.70)** with the DIFFICULTIES WITH DAY-TO-DAY ACTIVITIES domain scores of the EDQ.

EDQ Domain	N	Spearman Correlation	LB	UB	p-value	Hypothesis confirmed? Yes /No
Severity Daily	359	0.7	0.65	0.75	<0.001	No
Presence Daily	359	0.69	0.63	0.74	<0.001	No

Hypothesis 15-16*
Getting Along WHODAS summary scores will **moderately correlate (>0.50)** with the SOCIAL inclusion domain scores of the EDQ.

EDQ Domain	N	Spearman Correlation	LB	UB	p-value	Hypothesis confirmed? Yes /No
Severity Social	359	0.79	0.75	0.83	<0.001	No
Presence Social	359	0.73	0.68	0.78	<0.001	Yes

Hypothesis 7-18*
Life-Activities (Household) WHODAS summary scores will **strongly correlate (>0.70)** with the DIFFICULTIES WITH DAY-TO-DAY ACTIVITIES (Daily) domain scores of the EDQ.

EDQ Domain	N	Spearman Correlation	LB	UB	p-value	Hypothesis confirmed? Yes /No
Severity Daily	359	0.75	0.7	0.79	<0.001	Yes
Presence Daily	359	0.74	0.69	0.79	<0.001	No

Hypothesis 19-20*
Life-Activities (Work) WHODAS summary scores will **moderately correlate (>0.50)** with the SOCIAL inclusion domain scores on the EDQ.

EDQ Domain	N	Spearman Correlation	LB	UB	p-value	Hypothesis confirmed? Yes /No
Severity Social	156	0.64	0.53	0.72	<0.001	Yes
Presence Social	156	0.68	0.59	0.76	<0.001	Yes

Hypothesis 21-22*

Participation WHODAS summary scores will **strongly correlate (>0.70)** with the SOCIAL inclusion domain scores on the EDQ.

EDQ Domain	N	Spearman Correlation	LB	UB	p-value	Hypothesis confirmed? Yes /No
Severity Social	358	0.83	0.79	0.86	<0.001	Yes
Presence Social	358	0.8	0.76	0.83	<0.001	Yes

Hypothesis 23-24*

Participation WHODAS summary scores will **moderately correlate (>0.50)** with the MENTAL-EMOTIONAL domain scores on the EDQ.

EDQ Domain	N	Spearman Correlation	LB	UB	p-value	Hypothesis confirmed? Yes /No
Severity Mental Emotional	358	0.68	0.63	0.74	<0.001	Yes
Presence Mental Emotional	358	0.59	0.52	0.65	<0.001	Yes

Hypothesis 25-36* (Known Groups)

Participants who completed the EDQ on a 'good day' will have significantly lower EDQ domain scores (severity and presence) on the EDQ compared to those who completed the EDQ on a 'bad day'

Covariate	Full Sample (n=359)	Bad day (n=65)	Good day (n=294)	p-value	Hypothesis confirmed? Yes /No
**Severity Physical**				**<0.001**	Yes
Mean (sd)	32.1 (17.6)	45.4 (13.2)	29.1 (17.0)		
Median (Min,Max)	34 (0,77)	48 (0,71)	28 (0,77)		
**Presence Physical**				**<0.001**	Yes
Mean (sd)	53.2 (27.5)	72.2 (22.1)	48.9 (26.8)		
Median (Min,Max)	52 (0,100)	75 (0,100)	52 (0,100)		
**Severity Cognitive**				**<0.001**	Yes
Mean (sd)	25.9 (22.0)	42.4 (23.4)	22.2 (19.9)		
Median (Min,Max)	20 (0,100)	42 (0,100)	20 (0,100)		
**Presence Cognitive**				**<0.001**	Yes
Mean (sd)	58.9 (40.8)	82.6 (31.3)	53.7 (40.9)		
Median (Min,Max)	67 (0,100)	100 (0,100)	67 (0,100)		
**Severity Mental Emotional**				**<0.001**	Yes
Mean (sd)	37.9 (23.5)	59.0 (22.0)	33.3 (21.2)		
Median (Min,Max)	41 (0,100)	58 (0,100)	37 (0,100)		
**Presence Mental Emotional**				**<0.001**	Yes
Mean (sd)	66.1 (35.9)	88.9 (22.7)	61.1 (36.4)		
Median (Min,Max)	77 (0,100)	100 (0,100)	59 (0,100)		
**Severity Uncertainty**				**<0.001**	Yes
Mean (sd)	40.1 (20.9)	55.9 (20.4)	36.5 (19.4)		
Median (Min,Max)	39 (0,100)	55 (9,100)	35 (0,100)		
**Presence Uncertainty**				**<0.001**	Yes
Mean (sd)	71.4 (30.3)	85.5 (22.9)	68.2 (30.8)		
Median (Min,Max)	78 (0,100)	100 (22,100)	78 (0,100)		
**Severity Daily**				**<0.001**	Yes
Mean (sd)	21.2 (20.4)	33.8 (19.3)	18.4 (19.6)		
Median (Min,Max)	21 (0,100)	33 (0,100)	8 (0,89)		
**Presence Daily**				**<0.001**	Yes
Mean (sd)	46.1 (40.6)	69.1 (31.8)	41.0 (40.6)		
Median (Min,Max)	42 (0,100)	78 (0,100)	23 (0,100)		
**Severity Social**				**<0.001**	Yes
Mean (sd)	31.0 (18.4)	46.2 (13.0)	27.6 (17.8)		
Median (Min,Max)	34 (0,75)	46 (8,75)	27 (0,69)		
**Presence Social**				**<0.001**	Yes
Mean (sd)	51.3 (29.9)	72.6 (21.7)	46.6 (29.4)		
Median (Min,Max)	55 (0,100)	67 (18,100)	44 (0,100)		

Secondary (Exploratory) Hypotheses (n=44)

Hypothesis 37-38

PHQ-8 scores will **strongly correlate (>0.7)** with the MENTAL-EMOTIONAL symptoms and impairments domain scores of the EDQ (severity and presence scores)

EDQ Domain	N	Spearman Correlation	LB	UB	p-value	Hypothesis confirmed? Yes /No
Severity Mental Emotional	358	0.81	0.77	0.84	<0.001	Yes
Presence Mental Emotional	358	0.7	0.64	0.75	<0.001	No

Hypothesis 39-40

PHQ-8 scores will **moderately correlate (>0.5)** with the UNCERTAINTY domain scores of the EDQ (severity and presence scores)

EDQ Domain	N	Spearman Correlation	LB	UB	p-value	Hypothesis confirmed? Yes /No
Severity Uncertainty	358	0.67	0.61	0.72	<0.001	Yes
Presence Uncertainty	358	0.52	0.44	0.59	<0.001	No

Hypothesis 41-42

Note – higher scores on MOS-SSS indicate greater social support; and higher scores on EDQ indicate greater disability so we would expect negative Spearman Correlation s here.

Scores on the Social Support Scale will **not correlate (<0.3)** with the PHYSICAL domain scores of the EDQ

EDQ Domain	N	Spearman Correlation	LB	UB	p-value	Hypothesis confirmed? Yes /No
Severity Physical	357	-0.35	-0.43	-0.25	<0.001	Yes
Presence Physical	357	-0.3	-0.39	-0.2	<0.001	Yes

Hypothesis 43-44

Scores on the Social Support Scale will **not correlate (<0.3)** with the COGNITIVE domain scores of the EDQ

EDQ Domain	N	Spearman Correlation	LB	UB	p-value	Hypothesis confirmed? Yes /No
Severity Cognitive	357	-0.39	-0.47	-0.29	<0.001	Yes
Presence Cognitive	357	-0.38	-0.47	-0.29	<0.001	Yes

Hypothesis 45-46

Scores on the Social Support Scale will **weakly correlate (>0.3)** with the MENTAL-EMOTIONAL domain scores of the EDQ

EDQ Domain	N	Spearman Correlation	LB	UB	p-value	Hypothesis confirmed? Yes /No
Severity Mental Emotional	357	-0.46	-0.54	-0.37	<0.001	Yes
Presence Mental Emotional	357	-0.42	-0.5	-0.33	<0.001	Yes

Hypothesis 47-48

Scores on the Social Support Scale will **weakly correlate (≥0.3)** with the UNCERTAINTY domain scores of the EDQ

EDQ Domain	N	Spearman Correlation	LB	UB	p-value	Hypothesis confirmed? Yes /No
Severity Uncertainty	357	-0.33	-0.42	-0.24	<0.001	No
Presence Uncertainty	357	-0.23	-0.32	-0.12	<0.001	No

Hypothesis 49-52

Scores on the Social Support Scale will **not correlate (<0.3)** with the DIFFICULTIES WITH DAY-TO-DAY ACTIVITIES (Daily) domain scores of the EDQ

EDQ Domain	N	Spearman Correlation	LB	UB	p-value	Hypothesis confirmed? Yes /No
Severity Daily	357	-0.32	-0.41	-0.22	<0.001	Yes
Presence Daily	357	-0.32	-0.41	-0.22	<0.001	Yes
Severity Daily	357	-0.32	-0.41	-0.22	<0.001	Yes
Presence Daily	357	-0.32	-0.41	-0.22	<0.001	Yes

Hypothesis 53-56

Scores on the Social Support Scale will **weakly correlate (≥0.3)** with the SOCIAL domain scores of the EDQ

EDQ Domain	N	Spearman Correlation	LB	UB	p-value	Hypothesis confirmed? Yes /No
Severity Social	357	-0.48	-0.56	-0.4	<0.001	Yes
Presence Social	357	-0.47	-0.55	-0.38	<0.001	Yes
Severity Social	357	-0.48	-0.56	-0.4	<0.001	Yes
Presence Social	357	-0.47	-0.55	-0.38	<0.001	Yes

Hypothesis 57-68

Participants who rated their General Health status (self-reported) better will have lower EDQ scores (presence and severity) compared with those who rated their health status as fair or poor. **General Health scores will be weakly correlated (>0.30) with EDQ presence and severity scores.**

EDQ Domain	N	Spearman Correlation	LB	UB	p-value	Hypothesis confirmed? Yes /No
Severity Physical	357	0.63	0.56	0.69	<0.001	No
Presence Physical	357	0.56	0.49	0.63	<0.001	Yes
Severity Cognitive	357	0.48	0.39	0.55	<0.001	Yes
Presence Cognitive	357	0.46	0.37	0.54	<0.001	Yes
Severity Mental Emotional	357	0.49	0.41	0.57	<0.001	Yes
Presence Mental Emotional	357	0.44	0.35	0.52	<0.001	Yes
Severity Uncertainty	357	0.48	0.39	0.55	<0.001	Yes
Presence Uncertainty	357	0.36	0.27	0.45	<0.001	No
Severity Daily	357	0.61	0.54	0.67	<0.001	No
Presence Daily	357	0.61	0.54	0.67	<0.001	No
Severity Social	357	0.56	0.48	0.63	<0.001	Yes
Presence Social	357	0.53	0.45	0.6	<0.001	Yes

Hypotheses 69-80

Participants with 2 or more concurrent health conditions will have higher total EDQ presence scores and severity scores (n=353 participants)

These hypotheses were tested at the first assessment with a one-sided hypothesis test for a Spearman Correlation of COMTot and scale/domain total (Rasch) score greater than zero.

Score	Spearman	p-value	Hypothesis confirmed? Yes /No
Severity Physical	0.67	<0.001	Yes
Presence Physical	0.61	<0.001	Yes
Severity Cognitive	0.49	<0.001	Yes
Presence Cognitive	0.50	<0.001	Yes
Severity Mental Emotional	0.44	<0.001	Yes
Presence Mental Emotional	0.37	<0.001	Yes
Severity Uncertainty	0.45	<0.001	Yes
Presence Uncertainty	0.33	<0.001	Yes
Severity Daily	0.67	<0.001	Yes
Presence Daily	0.68	<0.001	Yes
Severity Social	0.54	<0.001	Yes
Presence Social	0.53	<0.001	Yes
